# From Nature to Innovation: The Uncharted Potential of Natural Deep Eutectic Solvents

**DOI:** 10.3390/molecules28227653

**Published:** 2023-11-18

**Authors:** Luísa Schuh, Marcella Reginato, Isadora Florêncio, Leila Falcao, Luana Boron, Eliana Fortes Gris, Victor Mello, Sônia Nair Báo

**Affiliations:** 1 Microscopy and Microanalysis Laboratory, Department of Cell Biology, Institute of Biological Sciences, University of Brasilia, Brasília 70910-900, Brazil; luisaschuh.vargas@gmail.com (L.S.); may.reginato@gmail.com (M.R.); isadoraflorenciofs@gmail.com (I.F.); victor.silva@unb.br (V.M.); 2 Cooil Cosmetics, Brasília 71070-524, Brazil; 3 Nanocycle Group, Brasília 72622-401, Brazil; 4 Inaturals SAS, 2 Bis, Impasse Henri Mouret, 84000 Avignon, France; leila.falcao@inaturals.fr; 5 Inaturals BR, Rua Gerson Luís Piovesan 200, Concórdia 89701-012, Brazil; luaboron@yahoo.com.br; 6 Department of Bromatology, Faculty of Ceilândia, University of Brasília, Ceilândia 72220-275, Brazil; elianagris@gmail.com

**Keywords:** NaDESs, green chemistry, biomimicry, green solvent, natural deep eutectic solvents

## Abstract

This review discusses the significance of natural deep eutectic solvents (NaDESs) as a promising green extraction technology. It employs the consolidated meta-analytic approach theory methodology, using the Web of Science and Scopus databases to analyze 2091 articles as the basis of the review. This review explores NaDESs by examining their properties, challenges, and limitations. It underscores the broad applications of NaDESs, some of which remain unexplored, with a focus on their roles as solvents and preservatives. NaDESs’ connections with nanocarriers and their use in the food, cosmetics, and pharmaceutical sectors are highlighted. This article suggests that biomimicry could inspire researchers to develop technologies that are less harmful to the human body by emulating natural processes. This approach challenges the notion that green science is inferior. This review presents numerous successful studies and applications of NaDESs, concluding that they represent a viable and promising avenue for research in the field of green chemistry.

## 1. Introduction

Nature has been adapting and changing for billions of years, affected by various factors such as evolution, climate change, biotic interactions, and other influences. Biomimicry methods have emerged to optimize efficiency and sustainability, particularly in engineering fields. This approach has also influenced other fields such as aerodynamics, robotic navigation, medicine, clothing design, and water pollution detection, demonstrating the potential to learn from nature’s capacity to adapt [1,2]. This opens a world of possibilities and technologies that can inspire innovation and products that are greener, more sustainable, and made with natural components [2].

One such innovative alternative is natural deep eutectic solvents (NaDESs), which have become a new green tool for improving drugs’ bioavailability and absorption, pharmaceuticals, nutraceuticals, and cosmetics. NaDESs comprise a group of green solvents resulting from a mixture of natural components, such as primary metabolites (amino acids, sugars, acids, and choline derivatives) that have a very low melting point, characterizing a eutectic mixture that presents varied properties. NaDESs are highly tunable and can be modified for specific goals. They are also used to reduce toxicity and lower the cost of processes in industries and companies while serving as a water-reducing alternative. Moreover, studies have shown that NaDESs are more biodegradable, less hazardous, and more stable than other eutectic solvents [3,4,5].

The features of NaDESs raise questions about how this technology can improve old processes and create new ones, particularly in fields like cryopreservation, cosmetics, the food industry, bioactive extraction, and nanocarriers. NaDESs can provide a new perspective and possibilities to explore in these fields, leading to better results and higher efficiency [6]. It is evident that NaDESs and biomimicry are linked and that this new class of solvent can be employed to reproduce solvent media found in nature, thereby directing it towards a new level of innovation and employability [7].

In summary, the application of biomimicry to NaDESs can lead to a vast resource of new possibilities and technologies that are more sustainable and environmentally friendly. The potential applications of NaDESs are numerous, and their use can lead to better results and increased efficiency in various fields. By learning from nature’s capacity to adapt, we can unlock a new level of innovation and create a better future for ourselves and the environment.

This review aims to analyze the trajectory of NaDESs, from their discovery to their application over the years, in addition to promoting a discussion about the different conceptions of this technology throughout the decades, the path of development since their forerunners, the relationship between NaDESs and biomimicry, and the wide range of applications that have been successfully explored during this journey. NaDESs’ benefits and the problems yet to be solved are also presented, discussing the improvements that can be made and their limitations. This review also challenges the erroneous but deeply rooted thinking that green chemistry is not viable, aiming to change this limited vision with the rising and outstanding technology of NaDESs.

## 2. Methodology

The approach used in this research was consolidated meta-analytic approach theory (TEMAC), a quantitative, exploratory approach with a multilingual model, which integrates several databases and uses only free programs as technological support (Table 1) [8].

TEMAC offers a wide variety of possibilities for interrelations and inferences on a given topic. This technique is capable of grouping universities, countries, and areas of knowledge, providing researchers with functionality and important information to identify resource needs and guide public policies. In addition, the effectiveness of TEMAC stands out for the time and cost involved, which are considered its main benefits. TEMAC analysis is carried out in clear steps and is based on bibliometric theories [8]. The choice of articles for the basis of this research used the flowcharts in Table 2 and Table 3, which show the fundamentals behind each of the methods used to choose articles.

This research was performed using two renowned databases, the Web of Science and Scopus. The result obtained by searching the keywords “natural deep eutectic solvents”, “natural deep eutectic solvent” and “NaDES”, which was 1.93% higher in Scopus. Of the total number of articles, 916 were found in both Scopus and Web of Science, returning a number of unique articles in each of the bases, 119 from Web of Science (with one less than the total, because it is a duplicated article) and 139 from Scopus.

Although the research was conducted using the search terms “natural deep eutectic solvents”, “natural deep eutectic solvent”, and NaDES, it is worth noting that the two databases yielded significant results related to this topic under different search terms. Therefore, a comprehensive survey was undertaken to enhance the research and to address methodological considerations.

One set of search terms employed for this investigation included “LTTMs” and “low transition temperature mixtures”. The search criteria for NaDES were replicated with the same timeframe of 2011–2023. In the Web of Science (WoS) database, 191 articles were identified, while in Scopus, 126 articles were retrieved. It is noteworthy that 82 of these articles were duplicated in both databases.

Subsequently, an additional search was conducted using the term “deep eutectic solvents”. Given that the terminology for NaDES is relatively recent and not universally standardized, many publications make reference to NaDES as deep eutectic solvent. This yielded a substantial number of results, with 7818 articles found in WoS and 10,229 in Scopus. Due to the volume of records, it was challenging to ascertain the exact number of duplicates.

In order to enrich this research, an examination was carried out on the 20 most-cited articles in each of the databases for each of the search terms. When focusing on the terms “LTTMs” and “low transition temperature mixtures”, only 3 articles were observed to be common to both databases. In contrast, with regard to deep eutectic solvents (DESs), 14 articles featured among the most cited in both WoS and Scopus.

Among the 63 articles cited in total, including both searches, 13 articles were identified in all three search categories: “LTTMs”, “low transition temperature mixtures”, and “deep eutectic solvents”. This resulted in 50 unique articles that were specific to each database and search term, thus avoiding duplication. It is pertinent to mention that out of these 50 unique articles, 9 were already cited in the references of this article.

Furthermore, it is noteworthy that among these 41 previously unexamined articles, many are authored by individuals who were already cited in the course of this research. A substantial portion of these unpublished articles represent more recent contributions by these same authors.

For information purposes, these 41 articles are contained in Supplementary Table S11.

By surveying the articles by the presented validation method, the presence of a writer named Nades with 71 articles published and stored on the Web of Science was noted. These articles were ignored during the presentation of data and choice of articles.

Table 2 presents each of the bibliometric principles involved in the analyses where we seek to know the data we are using. They are responsible for presenting us with arguments to interrelate the data and create filters [8].

Among the possibilities for using this model in science are discovering trends within one’s subject of study by analyzing the degree of significance of the subjects and how their behavior has developed over the years, ensuring the understanding of which areas are growing and which are losing publications and citations, in addition to areas that are unexplored or have a lack of studies [8].

To see the extensive analysis in choosing the articles that make up this review, Supplementary Materials can be requested.

## 3. NaDESs as They Are

Natural deep eutectic solvents (NaDESs) are composed of natural primary metabolites containing several hydroxyl groups, carboxyl groups, or amino groups, which facilitate hydrogen bonding interactions between the constituent molecules and form highly structured viscous liquids [9]. Green solvents are a generation of ecologically correct and more sustainable solvents that emerged with the aim of reducing the heavy environmental impacts caused by solvents derived from fossil resources [10,11]. To qualify as green, solvents must fulfill certain criteria such as availability, biodegradability, non-toxicity, recyclability, and low cost. Unfortunately, the number of solvents that meet these criteria is limited. Therefore, it is important to understand ionic liquids (ILs) and deep eutectic solvents (DESs) to comprehend the path to NaDESs [12].

Eutectic solvents were not widely reported in the literature until the early 2000s, with only a few studies reported in the 1990s that focused on specific applications of these liquid mixtures. Gill and co-workers reported in 1994 some eutectic mixtures as substrates for enzymatic reactions. This work demonstrated that when enzymes are dissolved in eutectic mixtures, they are able to retain their activity [13,14,15]. This proves that these mixtures are better reaction media than the conventional organic solvents. A work published in *Nature* in 1995 revealed the use of eutectic mixtures as a possible alternative for emulsion crystallization [13,16]. The Erbeldinger group reported in 1998 the use of heterogeneous eutectic mixtures for enzymatic synthesis that yield up to 80 water percentage recovery [13,17].

Studies on ILs date back to the early 20th century, and their initial main application was extraction. However, their use has shifted to synthesis, catalysis, and media for enzymatic reactions [18]. ILs consist mostly of ions, and therefore exhibit ionic conductivity. They include molten or fused salts with high melting points, as well as organic and inorganic salt eutectic mixtures with melting points or glass transition temperatures below 100 °C. The properties of ILs vary greatly, and modifications can be made to the cation and anion properties independently. They have higher liquid ranges than molecular solvents, and some slowly form glasses at low temperatures due to their negligible vapor pressures. The melting point of room-temperature ILs tends to decrease as the size and symmetry of the ions increases, and the density and viscosity of these liquids cover a wide range [19].

Despite their promising technical performance, studies have raised concerns about the toxicity and biodegradability of ILs. These issues include toxicity towards diverse organisms and ecosystems, high cost of synthesis and purification requirements, and potential environmental pollution through release via wastewater effluents [20,21].

However, in 2004, DES was first recognized as a versatile alternative for ionic liquids (ILs) [22]. DESs typically consist of two or three inexpensive and safe components that can associate with each other through interactions to produce a melting point lower than that of each individual component [18]. DESs also exhibit a significant decrease in the freezing point and are typically liquid at temperatures lower than 150 °C, with most DESs being liquid between room temperature and 70 °C [12].

DESs are widely recognized as a viable replacement for ILs due to the low cost of their starting materials, ease of preparation, and lack of waste generation. The most common DESs are based on choline chloride (ChCl) [22]. The research group of Wang et al. classified DES in three types: amide compounds, inorganic salts, and quaternary ammonium salts [23,24].

The physical properties of DESs, such as viscosity and conductivity, are determined by ion mobility and the availability of voids of suitable dimensions, which is consistent with the fluidity of other ionic liquids and molten salts. DESs have been found to improve upon some of the negative features of ILs, including lower toxicity and cost [22]. However, safety concerns have always been a major consideration among researchers. While DESs may offer a more sustainable alternative to many traditional ILs, they are not entirely sustainable due to the presence of metal salts in some formulations, which are known for their innate toxicity. Additionally, DESs are partly miscible with water, which can result in their release into the aqueous environment. It is worth noting that the components of DESs can be nontoxic and have low environmental impact [25].

As already mentioned above, NaDESs are composed of natural primary metabolites containing several chemical groups that facilitate the bonding between the molecules, forming a viscous liquid. These solvents can enhance their solubilization capacity by forming additional hydrogen bonds with solutes. NaDESs have a liquid-crystal-like structure in which all molecules are arranged in a matrix with optimal interactions via inter- and intramolecular hydrogen bonding [9]. However, when the water content exceeds 50% (*v*/*v*) or the solvent is diluted with water, the interactions weaken, leading to a decrease in viscosity. NaDESs can be tailored to have controllable physicochemical properties by adjusting the water content. For instance, the addition of 25% (*v*/*v*) water to the NaDES can decrease its viscosity almost to that of water. This property of NaDESs provides researchers with a basis from which to develop tailor-made solvents for specific applications [26]. While NaDESs hold promise as solvents for food, medicines, and cosmetics, further research is needed to fully explore their potential.

**Figure 1:**
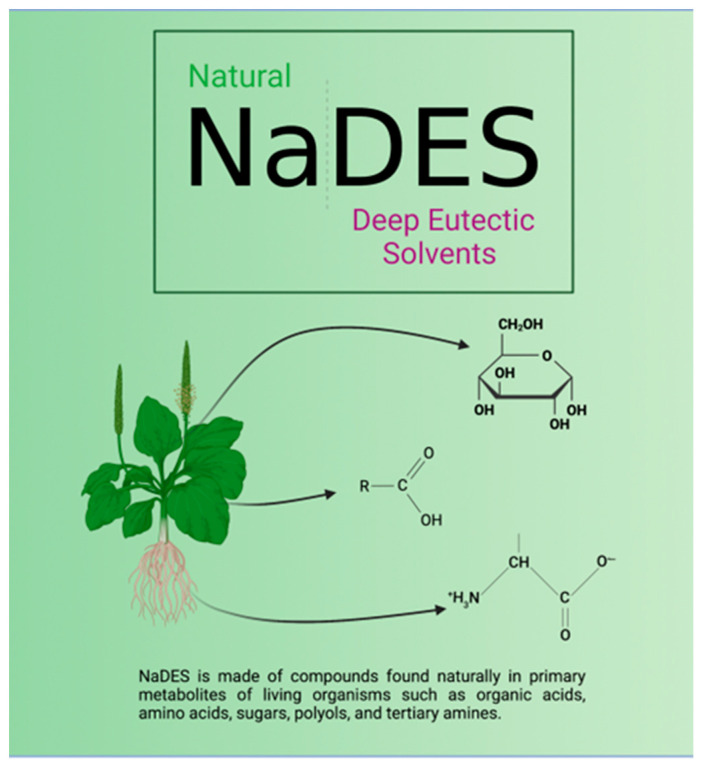
NaDESs are formed by the complexation of a hydrogen bond acceptor (HBA) and hydrogen bond donors (HBDs), similarly to DESs. This charge delocalization leads to a decrease in the melting point compared to that of the raw materials. NaDESs are made of compounds found naturally in primary metabolites of living organisms, such as organic acids, amino acids, sugars, polyols, and tertiary amines. The NaDES concept was developed to explain the higher solubility of certain natural compounds, such as flavonoids, than in water, working as a third liquid phase in organisms.

NaDESs are formed by the coordination entity of a hydrogen bond acceptor (HBA) and a hydrogen bond donor (HBD), similarly to DESs. This charge delocalization leads to a decrease in the melting point compared to that of the raw materials. The ease of preparing NaDESs, coupled with the vast number of possible combinations, makes them an attractive alternative to ILs for specific applications [13]. NaDES matrices are primarily determined by the intermolecular interactions among their components, allowing their properties to be easily influenced by various factors such as water content, temperature, and component ratio. This provides a challenge for the metabolomics of natural products but also presents an opportunity for tailor-made solvents with specific properties [27]. The supramolecular structure of NaDESs is held together by strong hydrogen networks, and loosening these networks could enhance their industrial applications [21] (Figure 1).

NaDESs emerged in the early 2000s when Abbott and co-workers classified DESs as a sub-family of ILs [28]. While ILs and NaDESs share some similarities such as a wide liquid range and tunability, NaDESs have unique characteristics that make them innovative. For example, NaDESs are less expensive, less hazardous, more biodegradable, more stable, and easier to synthesize on a large scale compared to ILs. Additionally, NaDESs are made of compounds found naturally in primary metabolites of living organisms, such as organic acids, amino acids, sugars, polyols, and tertiary amines [29]. These properties make NaDESs attractive solvents for various industries, and further research is needed to explore their full potential.

Some other studies have suggested that NaDESs could be a third liquid phase present in the cells of all living organisms, including plants [5]. The evidence for their presence is indirect, based on proton nuclear magnetic resonance (^1^H NMR) metabolomic analyses, which have shown high levels of common metabolites such as sucrose or glucose, with similar intensity signals in all types of biological material. This has led to theories that ILs could be present in plants due to their advantageous properties, such as the ability to biosynthesize non-water-soluble metabolites like cellulose. Natural ILs and DESs were identified after an experiment successfully mixed equimolar amounts of malic acid and ChCl and were named NaDESs. While the direct evidence of their presence in nature is still lacking, the amount of indirect evidence is increasing [18].

Indirect evidence suggests that drought, desiccation, and cold resistance are related to high levels of osmolytes, which encompass all the compounds found to form NaDESs. It is believed that a dynamic equilibrium exists between the aqueous phases of different cell compartments and the potential presence of NaDESs attached to membranes or in vesicles. Different NaDESs may exist in the same cellular space, attached to different membranes or in different compartments, highlighting the importance of water in their composition. The effect of water content on NaDESs’ solubilizing capacity and viscosity reduction has been studied, with dilution with water shown to activate certain enzymes in NaDES solutions and verify the protective role of NaDESs for proteins [18].

A study has demonstrated the impressive solubilizing capacity of NaDESs for a broad range of metabolites with low to medium polarity, as well as macromolecules like DNA, proteins, and polysaccharides. This capacity is attributed to the supramolecular structure of NaDESs and their broad range of polarity, which covers a spectrum from more polar than water to that of methanol [30]. The addition of small amounts of water decreases NaDESs’ viscosity significantly without compromising their unique characteristics. In human and mouse cell lines, NaDESs’ cytotoxicity seems to be related to various factors, such as the physical properties of NaDESs (particularly viscosity), the cellular requirements of cancer cells, and the nature of NaDESs’ raw materials and their interactions with functional groups present on the cell surface. Recent studies have also shown that the inclusion of organic acids in NaDESs can increase their overall toxicity. Furthermore, the Hofmeister phenomenon suggests that NaDESs/DESs species’ propensity to permeate cellular membranes depends on the specific principle of affinities between chaotropic and kosmotropic species and cell surface groups [21].

NaDESs have the potential to be used as drug delivery systems for poorly soluble bioactive compounds and are being considered as an alternative to traditional volatile organic solvents in various chemical processes and applications. In recent years, they have been utilized as solvents for carbon dioxide capture, biodiesel production and processing lignocellulose, biocatalysis, and electrochemical detection of phenolics. Moreover, NaDES extraction has been combined with microwave- and ultrasound-assisted techniques, innovative extraction technologies that reduce the energy and time required and increase extraction yields. The food, biomedical, cosmetics, and pharmaceutical industries are some of the sectors where NaDESs have shown significant promise [31].

## 4. NaDESs: Learning from Nature

Biomimicry, as defined by the Cambridge dictionary, is “the practice of designing technological and industrial products that copy natural processes” [32]. By employing biomimicry thinking, we can create bio-inspired products and overcome technical challenges in science, such as using NaDESs for cryopreservation or combining them with nanocarriers to improve drug delivery [33]. A NaDES is, in fact, a biomimicry of nature, since it is made up of natural components found in living organisms or in high concentrations during stressful situations. This natural origin of NaDESs provides numerous benefits when applied in product formulation or technology development, as previously discussed. NaDESs are, therefore, a new technology that enables us to learn from nature once again [29].

As mentioned earlier, there is evidence of the presence of NaDESs in living organisms. The first evidence that NaDESs may exist in nature was obtained in an investigation of highly concentrated molecules in saps, nectars or plant exudates. The strongest theories that supported this investigation were that the visual appearance and physical properties of these secretions and NaDESs are very close, their qualitative analysis usually showed the presence of molecules encountered in NaDES mixtures, and the stoichiometry between components are very consistent and close to the ones observed in NaDESs. These hypotheses were based on the nectar of the plant *Cleome hassleriana*, honey or maple syrup, in all of which were found compositions very similar to the ones found in NaDESs, with practically equimolar ratios [29].

NaDESs can be also seen as an explanation for unclear metabolic pathways in living organisms. This idea surrounds the thinking that many metabolites that are synthetized, stored and transported in plants are highly concentrated and sometimes are non-water-soluble. NaDESs, in this case, can be viewed as the medium in which the enzyme-catalyzed reactions of poorly water-soluble molecules or polymers would occur. The fact that NaDESs have an excellent capacity for solubilizing these substances and an unexpected activity/stability of enzymes in these media supports this statement. For example, other studies have shown that, in comparison with water, the increase in solubility of molecules such as rutin and quercetin ranged from 18 to 460.000 [29,30].

NaDESs form hydrogen bonds with surface residues of the enzyme, leading to stability instead of enzyme denaturation [29,34]. Moreover, some enzymes have been shown to be inactive in NaDESs. However, with laccases, for example, the addition of water activates them, which supports an idea of the role of NaDESs in preserving enzymes in plant seeds. In other words, this just supports the fact that NaDESs can be the media in which cell enzymes or substrates are stored independently or simultaneously dissolve to allow biochemical reactions [5,29,35].

Another contribution of NaDESs would be to allow organisms to resist or acclimate in environmental conditions classified as harsh (cold or drought environments). The self-organizing structure found in NaDESs allows the liquid components to remain in this state and stable over significant temperature ranges. NaDESs could also be seen as the way to prevent water crystallization inside living cells for better winter survival. This could be stated based on the fact that the interactions between the NaDES network and water are strong enough to maintain water liquid even at temperatures below 50 °C [29]. Other studies have also showed that components frequently described in NaDESs are produced, stored, and used by cell organisms and tissues under certain specific conditions [29,36] The insect *Nemoura arctica* is a clear example of this hypothesis: its glycerol increased by three orders of magnitude in cold acclimation [29,37]. The same observation was obtained with beetles from central Europe. The idea of NaDESs involving water molecules could also be a strategy for organisms, such as cacti, to hold water. It was observed that when there is water in the NaDES, it is strongly retained in the liquid and cannot evaporate easily. This could prevent total desiccation [5,29].

NaDESs’ physical-chemical properties have also exhibited great potential in stabilizing the environment for solutes. They decrease the movement of solute molecules, reducing contact at interfaces and, consequently, oxidative degradation. Natural pigments, for example, are very poorly stable in water, while they are stable in plants. For example, the carthamin pigment shows a higher stability in NaDESs than in water. This creates the hypothesis that NaDESs may stabilize this safflower pigment as well as other pigments [9,29]. NaDESs could also play a role in cellular functions by being a good medium in which to solubilize and stabilize RNA and DNA structures [29,38]. All these examples of the role of NaDESs in living beings and biochemical processes only justifies how we still have a lot to learn by observing natural processes. Practicing biomimicry is essential to the path of green chemistry and more sustainable processes.

Over 100 combinations of NaDESs have been generated by exploring various combinations of these common metabolites, which are abundantly present in all types of cells and organisms [30]. Studies from 2018 described at least 174 NaDES species [27], with approximately 108 possible combinations estimated. Green technology is a critical issue for preserving the environment and reducing negative human impact. Green technology promotes the use of non-hazardous media and the development of environmentally acceptable solubilization techniques that control the physical properties of media and develop new green solvents [30]. NaDESs can provide a clear and direct link to the development of new technologies and sustainability because of their unique advantages, including biodegradability, sustainability, low cost, and simple preparation, surpassing all the other solvents discussed in this article [26,39].

The term “eutectic” in the name NaDES refers to a mixture of components that, in specific proportions, have the lowest melting point, as mentioned previously. DESs can overcome the significant drawbacks of common ILs and share properties with NaDESs, such as biodegradability and low toxicity. DESs are obtained by simply mixing two or three “safe” components capable of forming a eutectic mixture. The NaDES concept was developed to explain the higher solubility of certain natural compounds, such as flavonoids, than in water, working as a third liquid phase in organisms. This observation, along with the diversity of redundant metabolites in natural resources and the occurrence of natural eutectic mixtures, influenced the concept [5].

In some NaDES formulations, water can be added to reduce viscosity and increase the polarity and extraction efficiency of phenolics. However, excess water can decrease extraction efficiency, weakening or breaking the intermolecular hydrogen bond structure of NaDES components and reducing the extractability of less polar components. The addition of water increases NaDESs’ conductability but weakens hydrogen bonding interactions between components. NaDESs also enhance the stability of natural compounds during extraction and storage [40].

As already discussed, NaDESs’ physicochemical properties are tunable by adjusting the HBA and HBD. Depending on the molar ratio and the starting components, factors like viscosity, solvency power, and stabilizing capability can be altered accordingly. Molar ratio has a critical impact on the hydrogen bond network and, hence, the polarity and viscosity [12]. When compared, the polarity of betaine monohydrate-based NaDES is within the range of water and methanol [41]. As another example of these properties, it is known that organic acid-based NaDESs are more polar than water while sugar- and polyalcohol-based NaDESs are less polar, with polarities close to methanol [42]. It can be said that NaDESs exhibit solvent duality because they have lipophilicity and hydrophilicity, although many of them are polar. This shows how this solvent is capable of dissolving both polar and nonpolar compounds [39,42,43,44,45].

NaDESs’ physical-chemical properties have been tested and studied in various conditions, including temperature, water content, and different organic acids, due to their relevant characteristics [46]. NaDESs have demonstrated antimicrobial effects and antioxidative properties under specific conditions [47]. NaDESs represent a rising technology that aims to revolutionize the way a wide range of products and drugs are produced, by mimicking nature’s ability to manage solutes and media with molecules of primary metabolites, since the beginning of time. The biochemical and food industries have taken notice of NaDESs due to their numerous properties, as discussed earlier.

## 5. The Solvent Solution: New Solutions to All Your Problems

Solvents play a vital role in many industries, from personal care products and pharmaceuticals to pesticides, cleaners, and paints. Interestingly, despite their widespread use, people often fail to recognize their ubiquitous presence in the products they consume. In the cosmetics industry, solvents such as ethanol, ethyl acetate, and acetone are used to provide appropriate consistency for lotions, powders, shaving creams, and nail polish. Solvents like butyl acetate are used in healthcare industries to purify penicillin, while in the paint industry, glycol ether esters are added to spray paints to prevent them from drying in mid-air. These examples barely scratch the surface of the countless applications of solvents in our daily lives. In fact, even the simple act of adding sugar to water, the universal solvent, is an application of solvent technology [48,49].

Although solvents play a crucial role in many industries, their importance often still goes unnoticed. However, the use and disposal of solvents pose significant environmental and health risks. The vapors and mists of many solvents have a narcotic effect and can cause fatigue, dizziness, and even death at high doses. Improper disposal of these solvents can lead to contamination of water, air, and soil, affecting the lives of living beings that meet them. To address these issues, there is a growing need for developing green technologies that focus on creating new, environment-friendly solvents [48]. NaDESs and room-temperature ionic liquids are promising solvents that can meet both technological and economic demands. These green solvents not only eliminate concerns regarding environmental impact, but also offer cheaper and simpler disposal options. Furthermore, using such solvents can free up resources that were previously allocated to proper disposal and redirect them towards other more productive areas. In conclusion, the development of green solvents such as NaDESs can address many of the challenges posed by traditional solvents while satisfying safety, effectiveness, and environmental restrictions [48,50].

The improper disposal of by-products generated by various industries, particularly the food industry, is a growing concern. These by-products are often considered a waste rather than a valuable source of compounds that could be used for various technological or nutritional purposes [51]. One promising solution to this problem is the use of NaDESs as solvents for the extraction of useful compounds from food by-products, such as anthocyanin and pectin from *Myrciaria cauliflora* fruit by-products (popularly known in Brazil as jabuticaba). Different natural food ingredients have been evaluated as substitutes for synthetic additives due to their health benefits [52]. This is possible due to the nutritional and technological aspects of these ingredients and, since Brazil has a large variety of fruits that generate co-products with valuable nutrients and bioactive compounds, Brazilian flora are gaining attention. These by-products have also been shown to be similar in composition to the intact vegetal matrix [53]. In recent studies, six NaDESs were prepared and characterized via various techniques, including differential scanning calorimetry, pH, and rheological analysis [54].

The affinity between NaDESs and the targeted molecules of the jabuticaba fruit was studied using COSMO-RS (conductor-like screening model for real solvents). The results show that the NaDES chlorine of chloride:proline (with a molar ratio of 1:2 and 1:1 in a water solution) was the most effective for anthocyanin extraction, while citric acid:glucose:water (with a molar ratio of 1:1:3 and 1:9 of NaDES/water in a water solution) was the most promising for pectin extraction. The efficiency of the NaDESs for anthocyanin and pectin extraction was evaluated using the maceration method and compared to various reference solvents, including ethanol, acidified ethanol, pure water, and citric acid. These results demonstrate that NaDESs are highly effective in extracting compounds that can be used in food applications and offer the added benefit of being eco-friendly. Furthermore, the selection of NaDES starting constituents can be tailored to different vegetable matrices and target compounds, making them versatile and widely applicable [54].

Ammonia (NH_3_) is a pollutant hazardous gas that exists mainly because of industrial processes [55]. Ammonia synthesis, the manufacturing of nitrogen fertilizer, metallurgy, and pharmaceutical industries are the biggest examples of how this gas is produced, used in everyday life and, consequently, affects the surroundings [56]. The direct emission of ammonia into the atmosphere may harm both the human body and the environment and, thus, separation and recovery of this gas from industrial exhaust gas are of great importance for pollution control and resource utilization [57]. In industry, the elimination of ammonia is normally carried out via the wet scrubbing method. Liquid solvents are usually used (water or aqueous inorganic acids), but these solvents are associated with some defects, such as high volatility and heat capacity. These characteristics can make the process of recycling NH_3_ environmentally unfavorable and energy intensive, particularly when it comes to the inorganic acids, which can exhibit strong corrosive qualities and reactivity, which makes the operation expensive and the recycling of ammonia quite difficult. A NaDES made with choline chloride and sugars was proposed, and it was more effective in capturing ammonia from dilute sources at elevated temperatures and low pressures when compared to other commonly used solvents. Because they enable strong hydrogen bond interactions with NH_3_, the abundant hydroxyl groups in NaDESs proved to be responsible for the highly efficient absorption of ammonia. Moreover, the absorption of NH_3_ in the NaDES displayed excellent selectivity over other industrial components present in tail gas and good reversibility [55]. Another study proposed a NaDES made with glycolic acid and xylitol (ratios 3:1) and it also displayed a higher ammonia absorption capacity that other ILs and DES tested previously and could be highly reversible under specific conditions. It showed similar selectiveness to NH_3_ as the aforementioned choline chloride and sugar NaDESs [57].

As discussed before, green solvents, especially DESs and NaDESs, are quite revolutionary and versatile, enabling applications in the most varied areas and, consequently, the resolution of many problems found with the use of traditional solvents. However, it is worth highlighting that, although they have strong potential as solvents, NaDESs and DESs have properties that can be understood as both advantages and disadvantages, depending on the application for which they are intended [45].

Among these properties, the high viscosity and low volatility of these solvents stand out. For example, while for the pharmaceutical and food industry, the high density and viscosity of these solvents can be considered an advantage, enabling better textures for formulations, for use in extraction, they hinder the interaction of the solvent with the substance, posing a challenge for industrial scaling. The same happens with low volatility, a property that makes these solvents more suitable for the environment while preventing their recyclability. Because of their low volatility, these solvents make film formation difficult, which creates a challenge for many applications [7,18,58].

Evidently, as with traditional solvents, there are other disadvantages associated with the use of green solvents, but it is worth analyzing whether, in fact, such characteristics only comprise disadvantages or whether they are simply being used for inappropriate applications.

Table 4 below presents a consolidated summary of the NaDESs discussed in the chapter with uses as solvents.

## 6. Small but Mighty: How Nanocarriers Are Revolutionizing Drug Delivery and the Role That NaDESs Play in This Story

Researchers are increasingly concerned about the environmental impact of scientific developments and are focusing on developing innovations with methods and materials that are biodegradable, biocompatible, and less toxic. This is also true for nanocarriers [59].

Nanoparticles are a type of manufactured material that measure less than 100 nm in at least two dimensions and present different properties because of their size, thus making their use an advantage compared to materials that are not in nanoscale. There is a variety of these particles, made from different kinds of materials and also using different methods, which enables their modifications for targeted molecular interactions [60].

Among their applications, nanoparticles are being used as nanocarriers in cancer drug treatments in order to promote more specific delivery. They are mostly made of synthetic polymers and not liquid-based carriers, which sometimes compromises certain uses [54]. However, researchers are now exploring new carrier systems using ILs and DESs, although these have limitations, such as thermal instability, low drug loading levels, and low drug release and solubility [61].

One possible solution to these issues is using NaDESs, which have been shown to be highly biocompatible materials that can transport drugs to a specific site without side effects. NaDESs can also be prepared using secondary metabolites, a method that has proven to be a significant achievement [62].

Studies have shown that NaDESs can be used as a biotin-conjugated solid–liquid nanocarrier (SLN) for the encapsulation of anticancer drugs, which can decrease cell toxicity caused by the drug. NaDESs made with lactic acid (LA) and prolinebetaine (PB) have shown promising results in the synthesis of the NaDES-based biotin-conjugated solid–liquid polymer nanocarrier through self-assembly. This method allows for the degradation of the drug carrier by lysosomal enzymes found in cancer cells, which helps to release drugs into cancer cells [54,61].

In another study using this system, the drug carrier contains both amide and ester bonds, with the amide bonds found in NaDESs facilitating the rapid degradation of the biotin-conjugated solid–liquid polymer by lysosomal enzymes in cancer cells [63]. This action aids the release of drugs specifically into cancer cells, providing a new perspective on drug site delivery and treatment effects. These results offer a wide range of possibilities for future experiments to improve treatment efficiency, while reducing side effects, targeting only the affected cells instead of healthy ones during cancer treatment [61].

While nanotechnology is increasingly used in commercial and industrial applications, its unique properties are also creating concerns [64,65]. Due to their large specific surface area and high reactivity, nanoparticles may bioaccumulate in organisms at the top of the food chain [66]. However, some studies have explored the use of nanoparticles in environmental and physiological applications, such as the extraction of metal oxides from plants using NaDESs [67].

To investigate this process, a study was conducted using radishes grown in a medium containing copper (II) oxide, cerium (IV) oxide, and titanium (IV) oxide. A NaDES was used as an extractant, and the results were analyzed using single-particle inductively coupled plasma mass spectrometry (SP-ICP-MS), since this technique can determine the number and size of the nanoparticles [68]. Interestingly, the copper (II) oxide nanoparticles were not found in the extract, regardless of the solvent used. Larger cerium (IV) oxide nanoparticles were found in the root, while smaller ones were present in the radish leaves. The titanium (IV) oxide nanoparticles were agglomerated and were present in small amounts in the plant leaves, but more accumulated in the root [67].

Various NaDES formulations were tested, and it was found that those containing choline chloride and either glucose or glycerol were the most effective at extracting metal oxide nanoparticles from plants without causing their transformation. However, it was also observed that a NaDES containing citric acid could not be used as an extractant because it dissolved nanoparticles of CuO [67].

Graphene has garnered significant attention for its potential applications in anti-cancer therapy, drug delivery, bio-imaging, and gene delivery. This molecule is non-toxic, and its cellular behavior has been confirmed as safe and biocompatible. It has a high surface area that benefits multiple attachment sites for drug targeting and has a drug loading capacity of up to a 200% loading ratio of loaded drug weight to delivery when compared with other drug nanocarriers [69]. It has already been applied as a nanocarrier for drugs such as ibuprofen, DOX (Doxorubicin), heparin, ellagic acid, 5-fluorouracil, and camptothecin [70,71,72]. However, there is a need to improve its biocompatibility, since it was speculated that this nanomaterial is potentially toxic to both humans and the environment [73]. In this study, a NaDES was used as a functionalizing agent [74].

NaDESs have been chosen because of their ability to introduce various functional groups and surface modifications. It is essential to modify the surface chemistry of graphene in order to achieve the goals surrounding the improvement of the biocompatibility. Among the NaDESs produced for the experiment, choline chloride:malonic acid (at a 1:1 molar ratio) proved to be the most efficient in reducing the cytotoxicity levels of graphene, while also demonstrating higher tamoxifen entrapment efficiency and loading capacity than other functionalized NaDESs. This high efficiency rate is due to the resulting surface modifications, including changes in morphology, exfoliation, re-stacking effect, and the addition of functional groups. The reduction in cytotoxicity was observed through various tests, including cell viability, cell cycle progression, and ROS generation. Overall, the use of NaDESs as a functionalizing agent has significant potential in enhancing the biocompatibility of graphene for various applications in medicine and beyond [74].

Therefore, while nanoparticles have many potential uses in commercial and industrial applications, their unique properties also present unique challenges. Thus, the use of NaDESs as an extractant for metal oxide nanoparticles in plants shows promise for further research in the environmental and physiological fields.

Researchers have also used NaDESs in the green cascade production of nanocellulose, nanohemicellulose, and nanolignin from *Prosopis juliflora* biomass, using NaDESs based on ChCl and lactic acid (LA), folic acid (FA), and oxalic acid (OA) in three different ratios: (1) 1:1; (2) 2:1 and (3) 3:1. Through screening experiments, LA2-ChCl was selected for selective hemicellulose solubilization in the first stage, and FA3-ChCl was selected for lignin solubilization in the second stage. This microwave-integrated two-stage cascade process gives a higher recovery yield (96.8% cellulose, 92.43% hemicellulose, and 90.56% lignin), and the recovered particles were then converted into nanoparticles using intense ultrasound. In summary, the use of NaDESs in this study for a proposed clean and sustainable process achieved the complete transformation of environmentally undesirable *Prosopis julifora* into sustainable nanoparticles and obtained maximum yield [75].

In another nanocellulose production study, this one using mechanochemical production coupled to NaDESs (made with ChCl and oxalic acid dihydrate or citric acid monohydrate), cellulose nanocrystals (CNCs) were extracted with a yield of 65%, and their lengths were established at around 143 nm. In addition, the carboxylated CNCs showed high thermal stability and a high crystallinity index, as this is a fast and direct method with little use of solvent, thus reducing the cost of producing CNCs and the negative environmental impact, promoting adaptation to the market for this renewable nanomaterial [76].

Another use of NaDESs in nanoscale productions involves the use of NaDESs based on ChCl and ascorbic acid (AA) to increase the solubility and antioxidant properties of antioxidant extracts from *Mangifera pajang* fruit residues, where all the ChCl-AA in different proportions tested increased the antioxidant capacity of antioxidant extracts by 1.3–14.64% compared to antioxidant extracts in water. This finding highlights the role of this NaDES as an antioxidant capacity enhancer, in addition to the antioxidant extracts solubilized in the ChCl-AA system, forming a nanoscale cluster structure, suggesting that it could potentially be used in a nanoformulation that protects antioxidant extracts [77].

Although these studies use NaDESs in processes that result in nanoscale formations, it is still hard to find reports in the literature of NaDESs being used for organic nanoparticles, especially lipid nanoparticles (LNs). LNs are nanoparticles that present at least one lipid in their formulations. They have emerged as a novel pharmaceutical drug delivery system of a variety of therapeutic agents, bringing the advantage of being biocompatible, biodegradable and less toxic. These particles can be classified in different types: solid lipid nanoparticle (SLN), nanostructured lipid carriers (NLCs), lipid drug conjugate (LDC), liposomes, and nanoemulsions [78,79].

Only one study reporting the use of NaDESs in lipid nanoparticles was found, more specifically with liposomes. The research aimed to investigate how NaDESs might affect skin permeation using liposome membranes of egg-phosphatidylcholine (Egg-PC) and di-palmitoyl-phosphatidylcholine (DPPC). The physical stability of liposomes was evaluated through their exposure to different NaDESs. The studies indicated that, in general, liposomes were physically stable in NaDESs for 24 h, being further stabilized by the solvent, in some cases. Furthermore, a decrease in liposome size was observed, which was attributed to the high osmolarity of the solvent [80].

However, regarding nanostructured lipid carriers or solid lipid nanoparticles, which are already used for cosmetic preparations, anticancer therapy and drug delivery, nothing related to NaDESs was found. The same happened with the other types of lipid nanoparticles.

Nonetheless, the scarcity of results does not mean that we cannot discuss and think about how the use of NaDESs in lipid nanoparticle formulations could solve some problems. Until now, we have mainly discussed NaDESs’ advantages, and if we stop to look at some points, a lot of these advantages can also be incorporated to improve lipid nanoparticle formulations.

For example, one of the biggest problems related to LN formulation is the use of toxic solvents to promote the solubility of some active compounds of interest to be loaded in the nanocarrier [81,82,83,84]. The use of NaDESs to try the solubilization of these active compounds could emerge as an innovative, green, and cheaper solution.

Another challenge commonly faced with LNs is their sensitivity to freezing conditions, which sometimes compromises the use of some protocols or even their storage for long periods [85,86]. Fortunately, some NaDESs are also known for their cryoprotective characteristics, and why should they not be tested for LN formulations?

Questions like these demonstrate the need for research in this area in order to increase the effectiveness of these studies and to revolutionize the production of green nanoparticles, adding value to the use of NaDESs. The applicability of NaDESs is vast, and there are endless possibilities for improvement, paving the way for a new wave of ideas [61].

Table 5 below presents a consolidated list of the NaDESs discussed in the chapter with uses in the area of nanoscience and nanotechnology.

## 7. NaDESs in Cryopreservation: Advancing Preservation Techniques

Over the last few decades, cryopreservation has become an area of great interest due to the increasing focus on fertility and optimizing organ transplants. However, the preservation of cells and tissues has its own set of challenges, with a significant portion of these challenges revolving around the viability of cells after the procedure. The cryopreservation of human ovarian tissue has been explored as a means of preserving the fertility of young women of fertile age before undergoing treatment for malignant disease. Similarly, these studies have applications in veterinary medicine, where female oocytes in ovarian follicles of endangered species, home pets, or transgenic animals can be preserved to build an animal germplasm bank. Ovarian follicles can be cryopreserved in two ways—in situ or isolated—each with its advantages, disadvantages, and challenges. In situ preservation is considered the most viable option, as preserving the ovary is difficult given the differences between the many cell types present in ovarian tissue [87,88,89].

**Figure 2:**
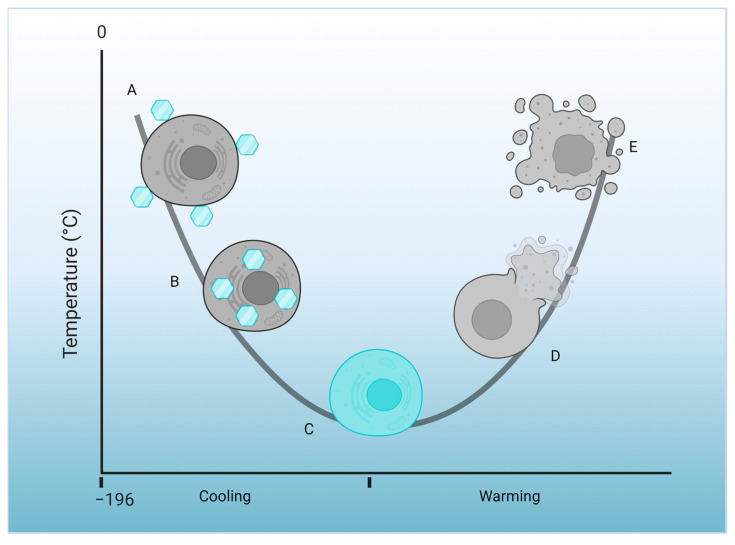
(A) Dehydration. (B) Supercooling and intracellular ice formation. (C) Vitrification. (D) Cell lysis. (E) Apoptotic onset. The use of NaDESs in cryopreservation can prevent the formation of extracellular ice crystals, avoiding osmotic imbalance and consequent cellular dehydration. Rapid cooling rates and the addition of high concentrations of cryoprotectant agents (CPA) can achieve vitrification, an amorphous and ice-free state. However, high concentrations of CPA can be toxic to cells. NaDESs exhibit low toxicity when compared to conventional cryoprotectants. Finally, cryopreservation can induce apoptosis, leading to delayed cell death after thawing. Due to their low toxicity, NaDESs can decrease the death rate.

Cryoprotectant agents, which are organic substances that protect cells or tissues against dehydration, cooling, and damage caused by extreme temperature reduction, play a crucial role in cryopreservation (Figure 2). The most commonly used cryoprotectants are dimethyl sulfoxide (DMSO), propanediol (PROH), ethylene glycol (EG), and glycerol (GLI). However, except for GLI, they are toxic to cells and tissues at specific temperatures, or in the case of PROH, during synthesis, they produce toxic intermediaries and pollutants [89,90].

When discussing the challenges of organ banking, it is imperative to address the obstacles related to ice nucleation and growth, cryoprotectant and osmotic toxicities, chilling injury, thermomechanical stress, the need for rapid and uniform rewarming, and ischemia/reperfusion injury. To overcome these challenges, various approaches have been proposed, including cryoprotectant screening strategies and the use of cryoprotectant cocktails that include ice-binding agents [91].

Several studies have shown that NaDESs exhibit anti-freezing properties and resistance to high osmotic pressure. These findings were observed in experiments that combined malic acid and choline chloride to create a homogeneous liquid mixture in a 1:1 ratio association. It was postulated that metabolites like choline or betaine, in combination with organic acids such as malic or citric, would create a liquid phase (NaDES) with “DES-like” physical-chemical properties that would account for these properties [29].

More recently, exciting results have been reported on the cryopreservation of Jurkat cells in NaDESs. An optimized cryoprotective agent (CPA) formulation was developed using a trehalose-glycerol NaDES (molar ratio of 1:30) diluted in Normosol-R and supplemented with isoleucine. The results show that the NaDES suppresses both ice formation and dehydration of the non-frozen region. Supplemented NaDES with isoleucine does not affect the solution’s thermophysical properties but significantly enhances the cells’ survival and proliferation post-thaw [33].

As a result, NaDESs are emerging as a potential substitute for cryoprotectants already used for cell cryopreservation, such as DMSO. NaDES is capable of reducing the number of ice crystals, thereby reducing ice crystal damage in cells, which is a critical factor in their survival upon freezing. Moreover, NaDESs are more biodegradable and less toxic than DMSO, the current gold standard for cryopreservation [85]. This opens up many possibilities, including the use of supplemented NaDESs to improve cell viability and survival after cryopreservation, enhancing the results and studies made with them.

Other studies conducted with L929 and HaCaT cells using a NaDES made of trehalose, glucose, proline, and sorbitol (in most systems, water was required to ensure the formation of the eutectic mixture) showed that the NaDES exerted a significant cryoprotective effect on L929 cells compared to DMSO or in the absence of a CPA. For HaCaT cells, the eutectic system demonstrated a slight improvement in cell survival, while DMSO caused complete cell death. In addition, the results obtained indicate that the NaDES could be maintained in the growth media after the thawing step without compromising cell viability, which is not possible with other CPAs such as DMSO [92].

The NaDES technology represents a significant innovation that could reduce or eliminate challenges with cryoprotectants in organ banking and fertility preservation. NaDESs are made with natural compounds that are minimally toxic and have a eutectic point with a low melting point, which could revolutionize the field of cryopreservation. The advantages of NaDESs are enormous, with the potential to extend the viability of organs for many days, months, or even years before transplant surgery. In fertility preservation, NaDESs could improve the viability of oocytes and other cells of the ovarian tissue, resulting in better preservation outcomes. Using NaDESs in this process of preservation would also decrease the amount of toxicity in the cryoprotectant, creating a less stressful environment for the cells. This would also reduce the number of toxic intermediaries and pollutants, thus contributing to the protection of nature and other living beings. Additionally, NaDESs use less water in these processes, improving water usage and reducing waste.

Table 6 below presents a consolidated list of NaDESs discussed in the chapter with uses in cryopreservation.

## 8. The Beauty of Science: The Fascinating Intersection of NaDESs, Cosmetics, Food, and Pharmaceuticals

NaDESs have emerged as an environmentally friendly option to replace petrochemicals in the cosmetics industry for dissolving plant metabolites. However, only a few NaDESs can be used for cosmetic purposes due to safety or regulatory issues [93]. NaDESs have also been studied for their potential in the food, pharmaceutical, and cosmetics industries, as demonstrated in a study analyzing anthocyanins in flower petals of *Catharanthus roseus* using HPLC-DAD-based metabolic profiling. The most promising NaDESs used in this study, including lactic acid:glucose (LGH), 1,2-propanediol:choline chloride (PCH), and 75% glucose:fructose:sucrose NaDES, showed similar extraction power for anthocyanins to acidified methanol, the preferred solvent in this process. LGH, in particular, exhibited at least three times the highest stabilizing capacity for cyanidins than acidified methanol, making it easier to extract and analyze these compounds [94].

Further research has demonstrated the excellent extractability of NaDESs for both polar and less polar metabolites, as seen in a study on safflower, a commonly used component in the food and cosmetics industry. NaDESs were found to be more effective at extracting metabolites than conventional solvents, with the water content in NaDESs having the most significant impact on phenolic compound yield. NaDESs successfully recovered most major phenolic compounds with a yield between 75% and 97%, making them a valuable option for extracting safflower, a plant used as a natural pigment, food additive, cosmetic, and traditional medicine for cardiovascular diseases [9,42].

NaDESs have also shown a high capacity for solubilizing and stabilizing carthamin, the primary red pigment in safflower, which has antioxidant activity but is very unstable in aqueous solutions. Unlike alkaline solutions, NaDESs do not cause degradation of carthamin, and they maintain its color and stability under various conditions such as high temperature, light, and storage time. NaDESs achieve this due to strong hydrogen bonding interactions between solutes and solvent molecules [9].

The awareness people nowadays have about their diet has led to a demand for industries to make food that contains biologically active compounds which have functional properties, such as antioxidant ones. Because of that, polyphenols are attracting attention, due to their potential positive effects in the area of human health [95]. Taking advantage of the high capacity of extractability of NaDESs, cocoa by-products were chosen as a sustainable source of polyphenols to be used as a fortifier of chocolate milk. The NaDESs used in this study and their molar ratios are: choline chloride:citric acid (2:1, ChCit), choline chloride:glycerol (1:2, ChGly), choline chloride:glucose (1:1, ChGlc), betaine:citric acid (1:1, BCit), betaine: glycerol (1:2, BGly) and betaine:glucose (1:1, BGlc). According to the results, the selected NaDESs could be used for efficient extraction of polyphenols from cocoa by-products, and the NaDES extracts obtained could be used for fortification in the food industry, without removal of extraction solvent, since they have been proven by tests to be safe and considered sensorially acceptable [96].

Bioactive compounds are widely used in both the cosmetics and food industries due to their many health benefits, including antioxidant, antimicrobial, antifungal, anti-inflammatory, anti-allergic, and antitumor effects [97,98,99]. They are used as replacements for synthetic additives, to confer bioactive characteristics, or to improve the sensory quality of food products. These compounds, such as phenolics, alkaloids, phenylpropanoids, terpenoids, polysaccharides, lipids, and peptides, are mostly extracted from natural matrices using aqueous-organic solvents like hexane, benzene, methanol, chloroform, petroleum ether, and acetone. However, these solvents can harm the environment, the operator, and consumer health due to their toxicity, volatility, and flammability [7].

Grapes are highly valued by the pharmaceutical, food, and cosmetics industries because of their potential applications. Grape phenolic compounds, including anthocyanins, flavanols, and phenolic acids, are known to have strong antioxidant [100,101,102], antimicrobial [103], anti-inflammatory [104,105,106], and anticancer [107] properties, as well as providing cardiovascular protection [108,109,110]. However, conventional methods used to extract grape phenolics are laborious, time-consuming, and require a huge amount of solvent use, often not delivering a good final product [39].

NaDES grape extracts have proven to be a promising alternative to conventional methods due to their simple, inexpensive, and naturally occurring compounds with an implied safety profile. In several tests, NaDES grape extracts were found to be of high quality with valuable biological activities, eliminating the need for expensive downstream purification steps [5,42]. However, due to the possible synergetic effect between components, it is necessary to further evaluate their cytotoxicity [111]. The cytotoxicity of five different types of NaDESs was evaluated in vitro using the cell lines MCF-7 and HeLa, and all of them formulated with 30% of water. The tested NaDESs were found to have low cytotoxicity, making them good options for extracting phenolics in grape skin, with choline chloride:fructose NaDES showing the best overall results [39]. In summary, NaDESs are a promising alternative to conventional extraction methods for bioactive compounds and have been shown to be particularly effective in extracting grape phenolic compounds. Further research is necessary to fully understand the potential applications and possible toxicological effects of NaDESs.

On the other hand, it is necessary to comment that there has been a research study in which NaDESs containing oxalic acid as a HBD showed the best performance in the phenolic extraction of grape skin. However, due to its toxicity, it was excluded from the study. This fact proves that many studies about NaDESs still need to be performed to understand why sometimes the best methodology for a specific application may not necessarily be the best one for the environment. Moreover, it also shows it is not always right to hypothesize that non-toxic raw materials will make a non-toxic solvent. That is why toxicity studies should always accompany their preparation and application. The extracts of the NaDES made with choline chloride:malic (ChMa) acid were tested for toxicity along with the other NaDESs, but only ChMa showed a substantial inhibiting effect on cell viability. It was evaluated via WST-1 cell proliferation assay using MCF-7 and HeLa. The methanol extract was also tested as a reference solvent for phenolic extraction. All the extracts were applied to cells in the range of 1.67 mg/mL and 16.67 mg/mL [39].

Recent research has shown that NaDESs based on a 30% solution of choline chloride:citric acid (molar ratio 1:1) are highly effective in the extraction of isoflavones from soy products [112]. Isoflavones are a group of chemical compounds found in plants known for their biological activity and are used in pharmaceutical formulations, dietetics, and the cosmetics industry [113]. Maintaining the proper level of isoflavones in the diet is important in preventing diseases such as cancer and cardiovascular diseases [112,114]. Classical extraction methods using significant amounts of solvents are laborious and require many samples. The NaDES used in these tests resulted in relatively non-toxic results and an easily prepared formulation made with renewable, non-flammable, non-volatile, and biodegradable components, optimizing extract efficiency. The optimized conditions for the procedure were a NaDES molar ratio of 1:1, 30% water content, a 3:1 NaDES volume to sample amount ratio, a 60 min extraction time, a 60 °C extraction temperature, and an ultrasonic power of 616 W. The results achieved enrichment factors up to 598 for isoflavones, with the recoveries of the analytes ranging from 64.7% to 99.2%. These findings suggest that NaDESs could be a promising alternative to traditional solvents for isoflavone extraction [112].

In the by-products field, some studies have been performing tests on NaDESs and microwave-assisted extraction (MAE) to recover bioactive compounds from hazelnut pomace (a by-product originating from the hazelnut oil process). The objective of this research is to contribute to sustainable valorization [40]. Research surrounding the choline chloride–propylene glycol mixture-based DES has already been performed and shows efficient properties in the extraction of flavonoids [115], anthocyanins [116], and phenolic compounds [117]. Trials were performed with eight different NaDESs, and choline chloride:1,2-propylene glycol (CC-PG, in the molar ratio of 1:4) was determined as the most suitable NaDES for the target extraction: in that case, the extraction of antioxidant constituents. The comparison and analyses established parameters for extraction efficiency and physicochemical properties. As already said, the physicochemical properties of NaDESs can be changed if the components’ hydrogen bond donors/hydrogen bond acceptor (HBD/HBA) structures or molar ratios vary. NaDES electrical conductivity is low, resulting in high viscosity, a factor that limits the effectiveness of NaDESs as solvents for extraction since the mass transporting capacity also decreases. While the physicochemical properties of the solvent are similar to water with the addition of 25% water (*v*/*v*), the solvent properties are preserved. It is known that NaDESs usually have high viscosity values at room conditions, but, in the results of this experiment, it was observed that the CC-PG NaDES and another one tested had much lower viscosity values, similar to the density data used as the base. Therefore, it is a positive feature in favorable extraction applications. The amounts of antioxidants extracted with three tested NaDESs, including CC-PG, were higher when compared to the other usual solvents, such as water, acetone, ethanol, and methanol. As already known, 1,2-propylene glycol is a nontoxic glycol preferred in formulations as a solvent in the pharmaceutical, cosmetics and flavoring industries. Although all these discoveries may be promising, it is still hard to predict the suitability of NaDESs by examining just the physicochemical properties [40].

As already mentioned, there is increasing interest in making use of food waste using environmentally friendly procedures, since it is known that the agri-food industry produces enormous amounts of waste. The olive oil industry produces residues such as twigs, leaves, and olive mill wastewater (OMW). Olive leaves are gaining interest for their use in high-added-value compounds. This part of the plant species *Olea europaea* L. contains large quantities of phenolic compounds, higher than those in the fruit or virgin olive oil [118]. Different from other plants, they have secondary metabolites, of which secoiridoid and flavonoids as the main ones. Oleuropein (Ole) is the most abundant bioactive phenol in olive leaf extract, having important pharmacological activities. Studies have described the anti-inflammatory properties of oleocanthal, since they are similar to ibuprofen [119]. After that, a group of pharmacological and biological activities were reported for this olive oil secoiridoid derivative and its analog, which is oleacein [120].

The main drawback in this context is the fact that only small amounts of either secoiridoid can be obtained from natural matrices. The conventional extraction of phenolic compounds from plant leaves and fruits is performed by maceration, also using organic solvents such as ethanol, methanol, dichloromethane, acetone, hexane, and ethyl acetate. Although the extraction process with these solvents has high yields and the product obtained is of high quality, these solvents harm human health and the environment. The process also requires long treatment times and high temperatures. Also, prior to use, they must be subjected to solvent removal and purification. As a result, it was seen that the combination of NaDESs and microwave-assisted extraction (MAE) techniques is an efficient approach to recover phenolic compounds from these wastes. The most effective NaDESs for this purpose were polyols-based, NaDES-2 (consisting of choline chloride and glycerol as HBD, as well as the most effective one), NaDES-4 (consisting of choline chloride and ethylene glycol), and organic acid-based NaDES-3 (consisting of choline chloride and lactic acid). It was also proved that NaDES extract could be fully used, even if applied to products used or consumed by people [118].

Phlorotannins (TPhCs) belong to the class of polyphenolic compounds and are secondary metabolites produced mainly by brown seaweeds (Phaeophyceae). They have diverse bioactivities, such as antiviral [121], anti-bacterial [122], antioxidant [123], anticancer [124], anti-inflammatory [125], neuroprotective [126] and UV-protective [127]. Due to their antimicrobial and antioxidant properties, phlorotannins are used in food packaging films as preservatives [128]. Also, they are co-extracted with fucoidan, providing cosmeceutical effects for fucoidan crude extracts [129]. In the European Union, phlorotannins are approved as new food products and are allowed as ingredients in dietary supplements [130]. NaDESs have been proposed as an alternative for the usually toxic solvents used for the extraction, such as ethanol (EtOH). The objective of the work of Obluchinskaya et al. was to study the effect of selected extraction parameters on the phlorotannin content, hydrophilic ascorbic acid and lipophilic fucoxanthin in a NaDES extract from *Fucus vesiculosus*. Two NaDESs were used: NaDES 1 was made with lactic acid and choline chloride (molar ratio of 3:1, respectively) and NaDES 2 was made with lactic acid, glucose, and H_2_O (molar ratio of 5:1:3, respectively). The addition of water in the NaDES led to a decrease in the viscosity and in the surface tension of the solvents, resulting in a positive impact on the mass transfer from the seaweed cells into the extract [30,131]. In NaDES 1, the best formulation had a water content of 30%, while in NaDES 2 the best formulation had a water content of three moles of water. The effects of the chosen NaDES and EtOH were monitored in phlorotannin extracts during storage for 360 days at the temperature of 25 °C. After 30 days, the TPhC in EtOH began to decline considerably faster compared to the TPhC in both NaDESs. Seventy percent of the phlorotannins degraded in EtOH after 360 days, while NaDES 1 enables greater stability of phlorotannins. The results suggest that NADESs could be considered as an alternative to the conventional techniques for the effective extraction of phlorotannins from *F. vesiculosus* with high antioxidant potential, since the NaDESs tested provided high stability and preserved the bioactivity of the extracts during storage [131]. This potential may have a relationship to their viscosity [9,131]. The dilution with water decreased the viscosity of NaDES 2 compared to NaDES 1. The higher the viscosity of the NaDES, the more negatively it will affect the movement of the molecules, which allows a stable interaction between the molecules of the NaDES components and the solutes. This results in reduced contact time for the metabolites on the NaDES surface with air, leading to a less oxidative degradation [94]. Oxygen also has a lower solubility in NaDESs than ethanol, a fact that helps to explain and understand the results [131,132].

It is already known that *Hibiscus sabdariffa* L. has natural pigments that are valuable to the food industry [133]. Associated, again, with the MAE technique, NaDESs with choline chloride as a base were made. Among several, the oxalic acid-based NaDES with choline chloride (molar ratio 1:1) had the best selectivity for anthocyanins and higher extraction yields of the bioactive compound, even better than the ones extracted with methanol. The potential of this particular NaDES for the extraction of these pigments is undeniable, due to its acidic profile. However, more studies are needed, since there were some problems with stability and recovery yield, for example [134].

Proteins have high importance in the food industry. In some studies, the glycation conditions of bovine serum albumin (BSA) with glucose were studied to optimize it with a NaDES, since this process usually takes a lot of time and requires high temperatures. The theory was that a NaDES could improve the grafting of glucose-glycated BSA by shifting the reaction equilibrium [135]. A NaDES was prepared based on other research methods [112,113], but with slight modifications. In short, this was a choline chloride:glucose NaDES in the molar ratio of 1:1. The obtained NaDES was also diluted in PBS to prepare different *w*/*w* solutions. It was also seen that a water content lower than 40% may limit the migration of reactant molecules, limiting the degree of graft. The results verify that the NaDES provided a non-aqueous medium to shift the reaction equilibrium cited before. Markers, sodium dodecyl sulfate-polyacrylamide gel electrophoresis (SDS-PAGE), and Matrix-assisted laser desorption/ionization time-of-flight mass spectrometry (MALDI-TOF-MS) analyses were made. It was seen that, when compared to water systems, the NaDES had more hydroxyl groups, more secondary structures in disorder, lower intrinsic fluorescence intensity, lower surface hydrophobicity, fewer thiol groups, higher absorption of visible UV, and higher emulsifying properties. The NaDES was recognized at the end as a promising solvent to increase the glycation extents and property changes of BSA, also promoting its functional activities, making it applicable to the food industry [43,135,136,137,138,139,140,141].

Searching for an increase in the shelf life of food, active packaging of extracts rich in bioactive compounds added to edible films has become a study focus [136]. In order to achieve this, a nontoxic solvent is needed that is also not harmful to the environment. Some researchers have tried to design NaDESs by lyophilization, targeting the extraction of anthocyanins from *Luma chequen*. Later, the NaDES would be added to an edible î-carrageenan film matrix; ultrasound-assisted extraction (UAE) was used, and with pH differential, the method was able to evaluate the anthocyanin content. The antioxidant capacity of extracts and the antibacterial capacity were evaluated via DPPH assay (2,2-diphenyl-1-picryl-hydrazyl-hydrate) and diffusion agar tests, respectively. In the end, it was observed that the glycerol-based NaDES was efficient in the role of anthocyanin extraction and that the extract inhibition had good results [134].

Other studies focused on preparing biodegradable active packaging with shrimp waste. In this case, the effort was put into the extraction of astaxanthin (ASX) using an ultrasound-assisted (UAE) NaDES, and the support of ASX-rich NaDES extracts to achieve the goal of the research. The ASX-NaDES extract obtained via the process, in optimum conditions (68.98 ± 1.22 mg ASX/g shrimp waste), was applied as a plasticizer for chitosan (CS)-based biodegradable films, which were prepared using just NaDES/CS. Since the extraction method with a NaDES was a success, it is now seen as a potential alternative instead of the traditional extraction and a good opportunity for the utilization of low-grade materials [137].

NaDESs have already shown antibacterial properties by themselves or in combination with photosensitizers and light. Some researchers are trying to combine the wound healing property of collagen and the antibacterial properties of NaDESs [43,138]. Citric acid, xylitol, and their respective aqueous dilutions were used in NaDESs for the tests and they all went through spectroscopic, calorimetric, and viscosity methods. It was seen that collagen exhibited variable unfolding properties dependent on the type of material and degree of the aqueous dilutions made in the NaDES and, in fact, the two types of collagens (telo- or atelocollagen) were susceptible to the process of unfolding when diluted in this solvent. It was verified in the results that the dissolving process of collagen happened in a highly diluted NaDES, showing similar results to when collagen is dissolved in acetic acid. This all resulted in the acknowledgment that NaDESs can dissolve collagen while maintaining the structural properties of the collagen, turning NaDESs into a potential excipient in collagen-based products. Synthesizing all the results gathered, collagen in a NaDES is more susceptible to molecular changes, which can be beneficial if one exploits the chemotactic properties via the attraction of cells involved in wound healing and the antibacterial properties of the combination of collagen and NaDES. An increased mechanical strength of freeze-dried collagen-NaDES sheets was observed, and a plasticizing effect of NaDES was seen when this solvent was at low concentrations. The combination seemed suitable for the development of a topical preparation in the future [139].

Some valuable products are seen as waste, as has already been discussed. In the case of durian seeds, valuable products could be processed from them, for example, seed gum and flour. Some tests have tried to combine NaDESs combined with gum in the form of eutectogel. Some outcomes demonstrated tunable characteristics, indicating that the properties of natural durian seed gum can be improved for specific applications in the future. Since the coated gel is edible, potentially low cost, and strongly sustainable, food preservation could be extended, increasing the commercial value [140].

Freezing is the most common technique for preserving food products. However, during practical production, which involves the freezing process of food or the frozen storage of these products, severe ice or frost that may accumulate in considerable amounts can affect production efficiency and food quality. These specific problems have held back the development of the frozen food industry, but in order to change that, researchers have explored the anti-freezing properties of NaDESs. NaDESs were made with the combination of proline:glucose (molar ratio of 5:3 and 1:1), proline:sorbitol (molar ratio of 1:1), and urea:glucose:calcium chloride (molar ratio of 3:6:1). The proline:sorbitol one in the molar ratio of 1:1 showed the best temperature sensitivity and frosting capacity after several tests, showing how NaDESs can be announced as an innovation in anti-freezing agents for industries, since they can be fabricated into functional materials for this purpose, like hydrogel and eutectogel [141].

It is known that the blueberry is a fruit rich in polyphenols, with great potential for the extraction of bioactive compounds. Most solvents used to extract these bioactive compounds need to be removed for the subsequent uses of these molecules. In order to study the gastro-protective effects and the biocompatibility of a blueberry crude extract (CE) obtained using a NaDES and the extraction fractions in a model of ethanol-induced gastric ulcer in rats, a NaDES containing choline chloride:glycerol:citric acid in the molar ratios of 0.5:2:0.5 was used. The animals were treated for 14 days with water, NaDES vehicle, CE, anthocyanin-rich-fraction (ARF), a non-anthocyanin phenolic fraction (NAPF), or lansoprazole (intragastric) before receiving the ethanol that would induce the gastric ulcer. In the end, it resulted in CE decreasing the ulcer index and preserving the integrity of the mucosa. The pretreatment with CE or ARF reduced glutathione depletion and the mucosa’s inflammatory response. The NaDES treatment, as well as the other treatments, reduced protein oxidation and nitric oxide overproduction in rats that were treated with ethanol. These outcomes suggest that a NaDES can obtain biocompatible extracts of this fruit without needing to remove them for use. In conclusion, the NaDES vehicle contributed to some protective effects, and it has been seen as a potential medium for obtaining extracts of blueberry that exhibit gastro-protective effects [142].

Studies are also applying NaDESs as a potential extractor of medicinal plants, such as *Sideritis scardica* and *Plantago major*, aiming to avoid organic solvents. This methodology is seen as an alternative to water–alcohol mixtures, plus the antimicrobial and genotoxic potential of the extract studied. The best extract results for total phenolic compounds were obtained using a NaDES prepared with choline chloride:glucose (molar ratio of 5:2) with the addition of 30% water. The extraction efficiency was calculated by measuring total phenolics and flavonoids. However, something unexpected happened: the extracts with this NaDES were inactive against all tested microorganisms. Meanwhile, extracts with the NaDES containing citric acid:1,2-propanediol (molar ratio of 1:4) and choline chloride:glycerol (molar ratio of 1:2) showed exciting activity against some microorganisms. All four NaDESs tested showed low genotoxicity, and cytotoxicity, and the extracts showed antimicrobial activity. Indeed, the results show that a NaDES can improve the effects of bioactive extracts. However, more studies are needed to fully understand the influence of the NaDES in question on the bioactivity of dissolved substances and, most importantly, the possibility of using these extracts in the pharmaceutical, food, and cosmetics industries, among others [143].

*Curcuma longa* L. is used worldwide as a spice and coloring agent, mainly in the textile, pharmaceutical, confectionery, and cosmetics industries [144]. Studies throughout the years have highlighted this component, which has been used in the medical treatment of many diseases because of its reported properties, such as antioxidant, anti-inflammatory, antibacterial, antidepressant, antidiabetic, and antitumor [145,146,147]. These properties have already been applied as a protective and preventive agent against cancer, AIDS, neurological, lung, liver, and cardiovascular diseases. In order to extract curcumin and antioxidants using the MAE technique, five NaDESs were prepared using binary combinations of choline chloride, lactic acid, fructose, and sucrose. The NaDESs with sucrose:choline chloride:water (molar ratio 1:4:4), fructose:choline chloride:water (molar ratio 1:5:5), sucrose:lactic acid:water (molar ratio 1:5:7), and lactic acid:choline chloride:water (molar ratio 1:1:2) exhibited higher total antioxidant capacity (TAC) and curcumin contents (CC) than those in an 80% methanol:water solvent, which is the one preferred in the literature. The NaDESs were characterized via Fourier transform infrared (FTIR) density and viscosity values analysis. Some essential parameters were studied in MAE. The MAE results show that this method has the potential to be an efficient and sustainable procedure in the natural dye, food and pharmaceutical industries, among others. The extracts can also turn into a ready-for-consumption product [144].

NaDESs are known as nonvolatile, but there are still a limited number of techniques capable of concentrating and isolating bioactive compounds or food components from them. In contrast, studies have demonstrated that NaDESs do not interfere with the analytical determination of solutes, opening up new possibilities. With that in mind, analytical characterization without time- and cost-consuming purification steps would be possible [31].

The plant named *Aralia elata* has medicinal properties in its roots, which are rich in biologically active natural products. Triterpene saponins constitute one of their major groups, and these metabolites are usually extracted by methanol and ethanol but, as already discussed, these two solvents present a wide range of problems associated with their use. NaDESs were recently proposed as promising alternative extractants for the isolation of natural products from medicinal plants and, in the study of Petrochenko and Orlova [148], triterpene saponins were successfully extracted from all the seven acid-based tested NaDESs. The 1:1 mixture of choline chloride and malic acid, as well as a 1:3 mixture of choline chloride and lactic acid, presented the highest efficiency achieved. It could be concluded that, for 13 metabolites, NaDESs were more efficient extractants in comparison with water and ethanol [148].

*Rhodiola rosea* L. is a plant used in traditional medicine as a food supplement due to its adaptogen properties [149,150,151,152]. More than 150 biologically active compounds have been identified in the rhizomes of the plant, such as flavonoids, phenyletanes and phenylpropanoids [151,153,154,155]. A NaDES made with lactic acid, glucose, fructose and water was used to extract phenyletanes and phenylpropanoids, which were analyzed afterwards by HPLC. The results show that the L-lactic acid:fructose-based NaDES could be considered a viable alternative to 40% aqueous ethanol for the extraction of salidroside, tyrosol, rosavin, rosin and cinnamyl alcohol (sum of phenyletanes and phenylpropanoids). To optimize the extraction, the Plackett–Burman design followed by the steepest ascent method was used. Even though this NaDES proved to be a good alternative for extracting the compounds, the recovery of tyrosol and cinnamyl alcohol was lower that the results with the usual solvent [131].

Also used in traditional and officinal medicine, the roots of *Glycyrrhiza glabra* L. (popularly known as licorice) have more than 400 biologically active compounds, among which triterpenoid saponins and flavonoid compounds dominate [156,157]. Medicinal plants can accumulate a high amount of trace elements due to the industrial load on the environment, which are transferred with solvents from the plant material to the extract [158,159]. In the work of Shikov et al., acid-based NaDESs (polar solvents) are seen as an alternative to co-extract trace elements from the roots of this plant [160,161,162]. Five NaDESs were used to co-extract trace elements and glycyrrhizic acid (GA, one of the key active principles in licorine and the dominant phytochemical in the plant) from the roots of *G. glabra* and, due to similarities in the pKa of tactic acid and GA, the yield of GA in lactic acid-based NaDESs was higher in comparison with the other NaDESs. In these NaDESs, the yield of GA even surpassed the yield of GA in water. The recovery of all elements (except Li) by all tested NaDESs was low, with values smaller than 6%. The metal pollution index, hazard quotient, hazard index and chronic daily intake were also calculated. The results of these parameters suggest that all tested NaDES extracts of *G. glabra* roots were non-toxic and should possess no potential health risk after both topical application and ingestion [162].

To better understand this review, Table 5 below was prepared, with the NaDESs mentioned throughout this work. It is important to note that there are NaDESs with the same components and in equal proportions for different purposes, showing the possible range of uses of these solvents.

The areas of cosmetics, food, and pharmaceuticals have a wide range of examples presented throughout the chapter. Table 7 below consolidates these examples, describing each of the NaDES presented, their molar proportions and areas of use.

## 9. Conclusions

This review highlighted how NaDESs have evolved, changed and been improved throughout the decades since their first studies. From there, the knowledge surrounding this new technology and the range of applications has become extensive and promising, although there is still much to discover, as has also been pointed out.

Based on the information presented, NaDESs are now being seen as an alternative to commonly used solvents, and they are already preferable to some others because they represent a sustainable alternative for industries, as well as having superior solvent properties than currently used ones. NaDESs have lower toxicity, require less water during production, and are safe for use and consumption. These properties make NaDESs a better alternative for companies to use in the formulation of new products, as they are inspired by the natural metabolites found in plants and cells, providing unique characteristics and improvements in various fields. NaDESs are highly tunable and have greater solubilization capacity, making them applicable in almost every field of knowledge. Additionally, the use of NaDESs can significantly decrease the amount of hazardous and toxic constituents people ingest or come into contact with, as they are made from natural components that our bodies already recognize.

The still recent nature of studies with NaDESs allows for the exploration of new ideas, formulations, and experiments in a wide range of fields. One promising application of NaDESs is their potential to act as powerful moisturizers that can reduce water loss in extreme environments and temperatures, which could have significant implications for various industries. NaDESs could also be used as cryoprotectants to preserve viable cells, organs, and oocytes for longer periods, raising the possibility of extending female fertility and creating a bank of organs for transplants; they could be associated with nanocarriers, reducing toxicity of drugs and improving drug site delivery; and they could be used to create natural supplements with higher bio-solubility, as well as much more.

Overall, we can learn a lot from nature and how it has adapted over billions of years. NaDESs are an example of how we can use biomimicry to create sustainable and eco-friendly products that can lead to significant advances in many fields. However, all these aspects show that there is still much to learn about NaDESs and their relationship with nature, but their potential for innovation and progress is promising. And, above all, we should be grateful for our ability to observe and learn from nature and use that knowledge to evolve and progress.

## Figures and Tables

**Table 1:** Research flowchart.

Step	Procedure	Details and Actions
1	Keyword identification	Search for articles using the keywords: “Natural Deep Eutectic Solvents”, “Natural Deep Eutectic Solvent”, or “NaDES”.
2	Time range specification	Define the study’s time frame: 2011–2023.
3	Database selection	Choose databases for article retrieval: Web of Science (1036 articles); Scopus (1055 articles).
4	Research data analysis in databases	Analyze articles using various criteria: Assess journals based on impact factor, h-index, and cite score; Identify journals with the most publications on the subject; Examine the topic’s evolution over time; Identify the most-cited documents; Identify the most-published authors; Determine the most-cited authors; Identify the countries with the highest publication output; Determine the universities with the highest publication output; Identify the agencies that fund the most research; Determine the areas with the most publications.
5	Article selection and validation	Conduct the following analyses for article selection: Examine keyword frequency; Perform co-citation analysis; Implement bibliographic coupling analysis; Investigate co-authorship patterns.
6	Finalize article selection and review	Read the abstract of each article and conduct a review for selection.

**Table 2:** Validation of articles through bibliometric laws [8].

Type of Bibliometric Filter	Law/Principle of Bibliometrics
Analysis of the most relevant magazines (Table S1a,b)	Bradford law, impact factor, and 80/20
Analysis of the journals that publish the most on the subject (Table S2)	Bradford law
Evolution of the theme year by year (Table S5)	Literature obsolescence and Goffman’s epidemic theory
Most-cited documents (Table S6a,b)	Law of elitism, 80/20 law, and quotes
Most-published authors (Table S3) vs. most-cited authors (Table S4)	Law of Lokta and law of elitism
Countries that published the most (Table S7)	80/20 law
Universities that published the most (Table S8)	80/20 law
Agencies that fund the most research (Table S9)	80/20 law
Areas that publish the most (Table S10)	80/20 law
Keyword frequency	80/20 law

**Table 3:** Comparative table between characteristics of ILs, DESs, and NADESs.

	IL	DES	NaDES
Formed by	Organic cation and organic/inorganic anion	Hydrogen bond acceptor (HBA) and hydrogen bond donor (HBD) (amide compounds, inorganic salts, and quaternary ammonium salts)	HBA and HBD from natural sources
Solubilization Ability	Good solubilizing capacity of a number of organic compounds	High	High solubilization for a wide range of metabolites with low to medium polarity, as well as macromolecules such as DNA, proteins, and polysaccharides
Extraction Ability	High	High	High
Formulation/Synthesis Facility	Low, demand solvent use	High, but higher melting points of many DES, however, can hamper their application as a green solvent at room temperature	High
Can be Recycled	Yes	Yes	Yes
Biodegradability	Mean	High	High
Toxicity	Toxicity towards diverse organisms and ecosystems	Some formulations may contain metallic salts, which are known for their innate toxicity	Low, but the inclusion of organic acids in NaDESs can increase their overall toxicity
ECO Friendly	Potential environmental pollution through release via wastewater effluents	Lack of waste generation, but not entirely sustainable due to the presence of metal salts in some formulations	Yes
Cost	High	Low cost of their starting materials	Low

**Table 4:** NaDES with uses as solvents.

NaDES	Molar Ratio	Possibility of Use
Chlorine of chloride:proline	1:2 1:1 (in water solution)	Anthocyanin extraction [54]
Citric acid:glucose:water	1:1:3 1:9 (in water solution)	Pectin extraction [54]
Chlorine of chloride:fructose:water	1:5:1	Ammonia absorption [55]
Chlorine of chloride:xylose:water	1:5:1	
Glycolic acid:xylitol	3:1	Ammonia absorption [57]

**Table 5:** NaDES involved in nanoscience area.

NaDES	Molar Ratio	Possibility of Use
Chlorine of chloride:glucose	Not showed	Extracting metal oxide nanoparticles from plants without causing their transformation [67]
Chlorine of chloride:glycerol	Not showed	
Choline chloride:malonic acid	1:1	Reducing the cytotoxicity levels of graphene, while also demonstrating higher tamoxifen entrapment efficiency and loading capacity [74]
Choline chloride:lactic acid	2:1	Selective hemicellulose solubilization [75]
Choline chloride:folic acid	3:1	Lignin solubilization [75]
Choline chloride:oxalic acid dihydrate	1:1	Extraction of cellulose nanocrystals with 65% yield, high thermal stability and high crystallinity index [76]
Choline chloride:citric acid monohydrate	1:1	
Choline chloride:ascorbic acid	1:1 1:2 2:1	Increasing the solubility and antioxidant properties of antioxidant extracts from *Mangifera pajang* fruit residues [77]

**Table 6:** NaDES with uses in cryopreservation.

NaDES	Molar Ratio	Possibility of Use
Choline chloride:malic acid	1:1	Anti-freezing properties and resistance to high osmotic pressure [29]
Trehalose:glycerol	1:30	Cryopreservation of Jurkat cells [33]
Trehalose:glucose:sorbitol:water	1:2:1:10	A significant cryoprotective effect on L929 cells compared to DMSO or in the absence of a CPA, and for HaCaT cells, demonstrated a slight improvement in cell survival, while DMSO caused complete cell death [92]
Glucose:proline:glycerol:water	3:5:3:21	
Betaine:trehalose:glycerol:water	2:1:3:7	
Betaine:trehalose:water	4:1:12	
Betaine:sucrose:proline:water	5:2:2:21	

**Table 7:** NaDES involved in the areas of cosmetics, food and pharmaceuticals.

NaDES	Molar Ratio	Possibility of Use
Lactic acid:glucose	5:1	Anthocyanin extraction [94]
Choline chloride:1,2-Propanediol	1:1 1:1.5 1:2 1:3	
Glucose:fructose:sucrose	1:1:1	
Choline chloride:citric acid	2:1	Polyphenol extraction [96]
Choline chloride:glycerol	1:2	
Choline chloride:glucose	1:1	
Betaine:citric acid	1:1	
Betaine:glycerol	1:2	
Betaine:glucose	1:1	
Choline chloride:fructose	1.9:1	Extracting phenolics in grape skin [39]
Choline chloride:citric acid	1:1	Extraction of isoflavones from soy products [112]
Choline chloride:propylene glycol	1:4	Extraction of flavonoids [115], anthocyanins [116], and phenolic compounds [117]
Choline chloride:glycerol	1:1	Extraction of phenolic compounds [118]
Choline chloride:ethylene glycol	1:1	
Choline chloride:lactic acid	1:1	
Lactic acid:glucose:water	5:1:3	Extraction of phenyletanes and phenylpropanoids [131]
Choline chloride:lactic acid Lactic acid:glucose:water	1:3 5:1:3	An alternative to the conventional techniques for the effective extraction of phlorotannins from *F. vesiculosus* with high antioxidant potential [131]
Choline chloride:oxalic acid	1:1	Selectivity for anthocyanins and higher extraction yields of the bioactive compound [134]
Choline chloride:glycerol	1:1	Anthocyanin extraction [134]
Choline chloride:glucose	1:1	A promising solvent to increase the glycation extents and property changes of BSA, also promoting its functional activities, making it applicable to the food industry [43,135,136,137,138,139,140]
Choline chloride:glycerol	1:2	Extraction of astaxanthin (ASX) using an ultrasound assisted (UAE) NaDES [137]
Choline chloride:oxalic acid	1:2	
Choline chloride:lactic acid	1:2	
Choline chloride:tartaric acid	1:2	
Choline chloride:malic acid	1:2	
Choline chloride:glycerol:citric acid	0.5:2:0.5	NaDESs can obtain biocompatible extracts of this fruit without needing to remove them for use. In conclusion, the NaDES vehicle contributed to some protective effects, and it has been seen as a potential medium for obtaining extracts of blueberry that exhibit gastroprotective effects [142]
Choline chloride:glucose	5:2	Potential extractor for the isolation of natural products from medicinal plants [143]
Choline chloride:sucrose:water	4:1:4	Curcumin and antioxidant extraction [145]
Choline chloride:fructose:water	5:1:5	
Sucrose:lactic acid:water	1:5:7	
Choline chloride:lactic acid:water	1:1:2	
Choline chloride:malic acid Choline chloride:lactic acid	1:1 1:3	Potential extractor for the isolation of natural products from medicinal plants [148]
Choline chloride:malonic acid Choline chloride:malic acid Choline chloride:tartaric acid Choline chloride:citric acid	1:1 1:1 2:1 1:1 (All DESs with 30% water addition)	Alternative for co-extracting trace elements from the roots of *Glycyrrhiza glabra* L. [160,161,162]
Lactic acid:glucose:water Choline chloride:lactic acid Choline chloride:malic acid	5:3:1 1:3 1:1	
Sucrose:citric acid Sorbitol:citric acid Sucrose:Lactic Acid Sorbitol:Lactic Acid Choline Chloride:Lactic Acid	3:1 3:1 3:1 3:1 1:3	

## Data Availability

Not applicable.

## Supplementary Materials

The following supporting information can be downloaded at: https://www.mdpi.com/article/10.3390/molecules28227653/s1, Table S1a: Most Relevant Journals (Web of Science); Table S1b: Most Relevant Journals (Scopus); Table S2: The Top 20 Publishers; Table S3: The Top 20 Authors; Table S4: The Top 20 Cited Authors; Table S5: Evolution of the Theme; Table S6a: Most Cited Documents (Web of Science); Table S6b: Most Cited Documents (Scopus); Table S7: Countries that Publish the Most; Table S8: Universities that Publish the Most; Table S9: Agencies that Provide the Most Funding; Table S10: Areas that Publish the Most on the Topic. Table S11: Most-cited articles when complementing the search with the terms “Low Transition Temperature Mixtures”, “LTTMs” and “Deep Eutectic Solvents.

## References

[1] Nkandu M.I., Alibaba H.Z. (2018). Biomimicry as an alternative approach to sustainability. Archit. Res..

[2] Pawlyn M. (2019). Biomimicry in Architecture.

[3] Faggian M., Sut S., Perissutti B., Baldan V., Grabnar I., Dall’Acqua S. (2016). Natural Deep Eutectic Solvents (NADES) as a Tool for Bioavailability Improvement: Pharmacokinetics of Rutin Dissolved in Proline/Glycine after Oral Administration in Rats: Possible Application in Nutraceuticals. Molecules.

[4] Płotka-Wasylka J., de la Guardia M., Andruch V., Vilková M. (2020). Deep eutectic solvents vs ionic liquids: Similarities and differences. Microchem. J..

[5] Choi Y.H., van Spronsen J., Dai Y., Verberne M., Hollmann F., Arends I.W.C.E., Witkamp G.J., Verpoorte R. (2011). Are Natural Deep Eutectic Solvents the Missing Link in Understanding Cellular Metabolism and Physiology?. Plant Physiol..

[6] Mehariya S., Fratini F., Lavecchia R., Zuorro A. (2021). Green extraction of value-added compounds form microalgae: A short review on natural deep eutectic solvents (NaDES) and related pre-treatments. J. Environ. Chem. Eng..

[7] Benvenutti L., Zielinski A.A.F., Ferreira S.R.S. (2019). Which is the best food emerging solvent: IL, DES or NADES?. Trends Food Sci. Technol..

[8] Mariano A.M., Rocha M.S. (2017). Revisão da literatura: Apresentação de uma abordagem integradora. AEDEM Int. Conf..

[9] Dai Y., Verpoorte R., Choi Y.H. (2014). Natural deep eutectic solvents providing enhanced stability of natural colorants from safflower (*Carthamus tinctorius*). Food Chem..

[10] Capello C., Fischer U., Hungerbühler K. (2007). What is a green solvent? A comprehensive framework for the environmental assessment of solvents. Green Chem..

[11] Clarke C.J., Tu W.C., Levers O., Bröhl A., Hallett J.P. (2018). Green and Sustainable Solvents in Chemical Processes. Chem. Rev..

[12] Zhang Q., De Oliveira Vigier K., Royer S., Jérôme F. (2012). Deep eutectic solvents: Syntheses, properties and applications. Chem. Soc. Rev..

[13] Paiva A., Craveiro R., Aroso I., Martins M., Reis R.L., Duarte A.R.C. (2014). Natural Deep Eutectic Solvents—Solvents for the 21st Century. ACS Sustain. Chem. Eng..

[14] Gill I., Vulfson E. (1994). Enzymic catalysis in heterogeneous eutectic mixtures of substrates. Trends Biotechnol..

[15] López-Fandiño R., Gill I., Vulfson E.N. (1994). Protease-catalyzed synthesis of oligopeptides in heterogenous substrate mixtures. Biotechnol. Bioeng..

[16] Davey R., Garside J., Hilton A., McEwan D., Morrison J. (1995). Purification of molecular mixtures below the eutectic by emulsion crystallization. Nature.

[17] Erbeldinger M., Ni X., Halling P.J. (1998). Enzymatic synthesis with mainly undissolved substrates at very high concentrations. Enzym. Microb. Technol..

[18] Vanda H., Dai Y., Wilson E.G., Verpoorte R., Choi Y.H. (2018). Green solvents from ionic liquids and deep eutectic solvents to natural deep eutectic solvents. Comptes Rendus Chim..

[19] Freemantle M., Welton T., Rogers R.D. (2009). An Introduction to Ionic Liquids.

[20] Magina S., Barros-Timmons A., Ventura S.P.M., Evtuguin D.V. (2021). Evaluating the hazardous impact of ionic liquids—Challenges and opportunities. J. Hazard. Mater..

[21] Hayyan M., Mbous Y.P., Looi C.Y., Wong W.F., Hayyan A., Salleh Z., Mohd-Ali O. (2016). Natural deep eutectic solvents: Cytotoxic profile. SpringerPlus.

[22] Abbott A.P., Boothby D., Capper G., Davies D.L., Rasheed R.K. (2004). Deep Eutectic Solvents Formed between Choline Chloride and Carboxylic Acids:  Versatile Alternatives to Ionic Liquids. J. Am. Chem. Soc..

[23] Wang J., Han J., Khan M.Y., He D., Peng H., Chen D., Xie X., Xue Z. (2017). Deep eutectic solvents for green and efficient iron-mediated ligand-free atom transfer radical polymerization. Polym. Chem..

[24] Gómez A.V., Biswas A., Tadini C.C., Furtado R.F., Alves C.R., Cheng H.N. (2019). Use of natural deep eutectic solvents for polymerization and polymer reactions. J. Braz. Chem. Soc..

[25] Smith E.L., Abbott A.P., Ryder K.S. (2014). Deep Eutectic Solvents (DESs) and Their Applications. Chem. Rev..

[26] Dai Y., Witkamp G.J., Verpoorte R., Choi Y.H. (2015). Tailoring properties of natural deep eutectic solvents with water to facilitate their applications. Food Chem..

[27] Liu Y., Friesen J.B., McAlpine J.B., Lankin D.C., Chen S.N., Pauli G.F. (2018). Natural Deep Eutectic Solvents: Properties, Applications, and Perspectives. J. Nat. Prod..

[28] Abbott A.P., Capper G., Davies D.L., Rasheed R.K., Tambyrajah V. (2003). Novel solvent properties of choline chloride/urea mixtures. Chem. Commun..

[29] Durand E., Villeneuve P., Bourlieu-lacanal C., Carrière F., Verpoorte R., Witkamp G.J., Choi Y.H. (2021). Chapter Six—Natural Deep Eutectic Solvents: Hypothesis for Their Possible Roles in Cellular Functions and Interaction with Membranes and Other Organized Biological Systems. Advances in Botanical Research.

[30] Dai Y., van Spronsen J., Witkamp G.J., Verpoorte R., Choi Y.H. (2013). Natural deep eutectic solvents as new potential media for green technology. Anal. Chim. Acta.

[31] Mišan A., Nađpal J., Stupar A., Pojić M., Mandić A., Verpoorte R., Choi Y.H. (2020). The perspectives of natural deep eutectic solvents in agri-food sector. Crit. Rev. Food Sci. Nutr..

[32] Cambridge Dictionary (2023). Meaning of Biomimicry in English.

[33] Hornberger K., Li R., Duarte A.R.C., Hubel A. (2021). Natural deep eutectic systems for nature-inspired cryopreservation of cells. AIChE J..

[34] Xu P., Zheng G.W., Zong M.H., Li N., Lou W.Y. (2017). Recent progress on deep eutectic solvents in biocatalysis. Bioresour. Bioprocess..

[35] Le Roy J., Huss B., Creach A., Hawkins S., Neutelings G. (2016). Glycosylation is a major regulator of phenylpropanoid availability and biological activity in plants. Front. Plant Sci..

[36] Yancey P.H. (2005). Organic osmolytes as compatible, metabolic and counteracting cytoprotectants in high osmolarity and other stresses. J. Exp. Biol..

[37] Walters K.R., Sformo T., Barnes B.M., Duman J.G. (2009). Freeze tolerance in an arctic Alaska stonefly. J. Exp. Biol..

[38] Mamajanov I., Engelhart A.E., Bean H.D., Hud N.V. (2010). DNA and RNA in anhydrous media: Duplex, triplex, and G-quadruplex secondary structures in a deep eutectic solvent. Angew. Chem. Int. Ed..

[39] Radošević K., Ćurko N., Gaurina Srček V., Cvjetko Bubalo M., Tomašević M., Kovačević Ganić K., Redovniković I.R. (2016). Natural deep eutectic solvents as beneficial extractants for enhancement of plant extracts bioactivity. LWT.

[40] Bener M., Şen F.B., Önem A.N., Bekdeşer B., Çelik S.E., Lalikoglu M., Aşçı Y.S., Capanoglu E., Apak R. (2022). Microwave-assisted extraction of antioxidant compounds from by-products of Turkish hazelnut (*Corylus avellana* L.) using natural deep eutectic solvents: Modeling, optimization and phenolic characterization. Food Chem..

[41] Zahrina I., Nasikin M., Krisanti E., Mulia K. (2018). Deacidification of palm oil using betaine monohydrate-based natural deep eutectic solvents. Food Chem..

[42] Dai Y., Witkamp G.J., Verpoorte R., Choi Y.H. (2013). Natural Deep Eutectic Solvents as a New Extraction Media for Phenolic Metabolites in *Carthamus tinctorius* L.. Anal. Chem..

[43] Wikene K.O., Rukke H.V., Bruzell E., Tønnesen H.H. (2017). Investigation of the antimicrobial effect of natural deep eutectic solvents (NADES) as solvents in antimicrobial photodynamic therapy. J. Photochem. Photobiol. B Biol..

[44] Liu Y., Zhang Y., Chen S.N., Friesen J.B., Nikolić D., Choules M.P., McAlpine J.B., Lankin D.C., Gemeinhart R.A., Pauli G.F. (2018). The influence of natural deep eutectic solvents on bioactive natural products: Studying interactions between a hydrogel model and Schisandra chinensis metabolites. Fitoterapia.

[45] Kua Y.L., Gan S. (2019). Natural deep eutectic solvent (NADES) as a greener alternative for the extraction of hydrophilic (polar) and lipophilic (non-polar) phytonutrients. Key Eng. Mater..

[46] Savi L.K. Desenvolvimento de Solventes Eutéticos Naturais Profundos (NADES) e o Estudo de Suas Propriedades Físico-Químicas, Térmicas e Reológicas. [Dissertation on the Internet]. Paraná (BR): Universidade Federal do Paraná. Setor de Tecnologia. Programa de Pós-Graduação em Engenharia de Alimentos; 2019. https://hdl.handle.net/1884/61414.

[47] Radošević K., Čanak I., Panić M., Markov K., Bubalo M.C., Frece J., Srček V.G., Redovniković I.R. (2018). Antimicrobial, cytotoxic and antioxidative evaluation of natural deep eutectic solvents. Environ. Sci. Pollut. Res..

[48] Cvjetko Bubalo M., Vidović S., Radojčić Redovniković I., Jokić S. (2015). Green solvents for green technologies. J. Chem. Technol. Biotechnol..

[49] Chemical Safety Facts (2022). Solvents.

[50] Häckl K., Kunz W. (2018). Some aspects of green solvents. Comptes Rendus Chimi..

[51] Schieber A., Stintzing F.C., Carle R. (2001). By-products of plant food processing as a source of functional compounds—Recent developments. Trends Food Sci. Technol..

[52] Rodriguez-Amaya D.B. (2016). Natural food pigments and colorants. Curr. Opin. Food Sci..

[53] Gurak P.D., De Bona G.S., Tessaro I.C., Marczak L.D.F. (2014). Jaboticaba Pomace Powder Obtained as a Co-product of Juice Extraction: A Comparative Study of Powder Obtained from Peel and Whole Fruit. Food Res. Int..

[54] Benvenutti L., del Pilar Sanchez-Camargo A., Zielinski A.A.F., Ferreira S.R.S. (2020). NADES as potential solvents for anthocyanin and pectin extraction from Myrciaria cauliflora fruit by-product: In silico and experimental approaches for solvent selection. J. Mol. Liq..

[55] Li Z.L., Zhong F.Y., Huang J.Y., Peng H.L., Huang K. (2020). Sugar-based natural deep eutectic solvents as potential absorbents for NH3 capture at elevated temperatures and reduced pressures. J. Mol. Liq..

[56] Fu H., Luo Z., Hu S. (2020). A temporal-spatial analysis and future trends of ammonia emissions in China. Sci. Total Environ..

[57] Liu B., Tian J. (2021). Investigation of glycolic acid natural deep eutectic solvents with strong proton donors for ammonia capture and separation. Ind. Eng. Chem. Res..

[58] Ruesgas-Ramón M., Figueroa-Espinoza M.C., Durand E. (2017). Application of deep eutectic solvents (DES) for phenolic compounds extraction: Overview, challenges, and opportunities. J. Agric. Food Chem..

[59] Hoang Thi T.T., Pilkington E.H., Nguyen D.H., Lee J.S., Park K.D., Truong N.P. (2020). The Importance of Poly(ethylene glycol) Alternatives for Overcoming PEG Immunogenicity in Drug Delivery and Bioconjugation. Polymers.

[60] Boisseau P., Loubaton B. (2011). Nanomedicine, nanotechnology in medicine. Comptes Rendus Phys..

[61] Sun X., Pradeepkumar P., Rajendran N.K., Shakila H., Houreld N.N., Al Farraj D.A., Elnahas Y.M., Elumalai N., Rajan M. (2020). Natural deep eutectic solvent supported targeted solid–liquid polymer carrier for breast cancer therapy. RSC Adv..

[62] Pradeepkumar P., Elgorban A.M., Bahkali A.H., Rajan M. (2018). Natural solvent-assisted synthesis of amphiphilic co-polymeric nanomicelles for prolonged release of camptothecin delivery. New J. Chem..

[63] Gheybi H., Entezami A.A. (2013). Nanosized micelles self-assembled from amphiphilic poly(citric acid)–poly(ε-caprolactone)–poly(citric acid) copolymers. Polym. Bull..

[64] Ghosh S.K., Pal T. (2007). Interparticle Coupling Effect on the Surface Plasmon Resonance of Gold Nanoparticles:  From Theory to Applications. Chem. Rev..

[65] Buzea C., Pacheco I.I., Robbie K. (2007). Nanomaterials and nanoparticles: Sources and toxicity. Biointerphases.

[66] Hayder M., Wojcieszek J., Asztemborska M., Zhou Y., Ruzik L. (2020). Analysis of cerium oxide and copper oxide nanoparticles bioaccessibility from radish using SP-ICP-MS. J. Sci. Food Agric..

[67] Jakubowska M., Ruzik L. (2021). Application of Natural Deep Eutectic Solvents for the metal nanoparticles extraction from plant tissue. Anal. Biochem..

[68] Laborda F., Bolea E., Jiménez-Lamana J. (2014). Single Particle Inductively Coupled Plasma Mass Spectrometry: A Powerful Tool for Nanoanalysis. Anal. Chem..

[69] Liu J., Cui L., Losic D. (2013). Graphene and graphene oxide as new nanocarriers for drug delivery applications. Acta Biomater..

[70] Bitounis D., Ali-Boucetta H., Hong B.H., Min D.H., Kostarelos K. (2013). Prospects and Challenges of Graphene in Biomedical Applications. Adv. Mater..

[71] Yang K., Feng L., Shi X., Liu Z. (2013). Nano-graphene in biomedicine: Theranostic applications. Chem. Soc. Rev..

[72] de Melo-Diogo D., Costa E.C., Alves C.G., Lima-Sousa R., Ferreira P., Louro R.O., Correia I.J. (2018). POxylated graphene oxide nanomaterials for combination chemo-phototherapy of breast cancer cells. Eur. J. Pharm. Biopharm..

[73] Chen K., Ling Y., Cao C., Li X., Chen X., Wang X. (2016). Chitosan derivatives/reduced graphene oxide/alginate beads for small-molecule drug delivery. Mater. Sci. Eng. C.

[74] Zainal-Abidin M.H., Hayyan M., Ngoh G.C., Wong W.F. (2020). Doxorubicin Loading on Functional Graphene as a Promising Nanocarrier Using Ternary Deep Eutectic Solvent Systems. ACS Omega.

[75] Jacob Rani B.S., Venkatachalam S. (2022). Cleaner approach for the cascade production of nanocellulose, nanohemicellulose and nanolignin from *Prosopis juliflora*. Carbohydr. Polym..

[76] Douard L., Belgacem M.N., Bras J. (2022). Extraction of Carboxylated Nanocellulose by Combining Mechanochemistry and NADES. ACS Sustain. Chem. Eng..

[77] Ling J.K.U., Chan Y.S., Nandong J., Chin S.F., Ho B.K. (2020). Formulation of choline chloride/ascorbic acid natural deep eutectic solvent: Characterization, solubilization capacity and antioxidant property. LWT.

[78] Tenchov R., Bird R., Curtze A.E., Zhou Q. (2021). Lipid Nanoparticles—From Liposomes to mRNA Vaccine Delivery, a Landscape of Research Diversity and Advancement. ACS Nano.

[79] Shah R., Eldridge D., Palombo E., Harding I. (2015). Lipid Nanoparticles: Production, Characterization and Stability.

[80] Nystedt H.L., Grønlien K.G., Tønnesen H.H. (2021). Interactions of natural deep eutectic solvents (NADES) with artificial and natural membranes. J. Mol. Liq..

[81] Duan L., Dou L.L., Guo L., Li P., Liu E.H. (2016). Comprehensive Evaluation of Deep Eutectic Solvents in Extraction of Bioactive Natural Products. ACS Sustain. Chem. Eng..

[82] Mbous Y.P., Hayyan M., Hayyan A., Wong W.F., Hashim M.A., Looi C.Y. (2017). Applications of deep eutectic solvents in biotechnology and bioengineering—Promises and challenges. Biotechnol. Adv..

[83] Tong X., Yang J., Zhao Y., Wan H., He Y., Zhang L., Wan H., Li C. (2021). Greener extraction process and enhanced in vivo bioavailability of bioactive components from *Carthamus tinctorius* L. by natural deep eutectic solvents. Food Chem..

[84] Jeliński T., Przybyłek M., Cysewski P. (2019). Natural Deep Eutectic Solvents as Agents for Improving Solubility, Stability and Delivery of Curcumin. Pharm. Res..

[85] Castro V.I.B., Craveiro R., Silva J.M., Reis R.L., Paiva A., Duarte A.R. (2018). Natural deep eutectic systems as alternative nontoxic cryoprotective agents. Cryobiology.

[86] Jesus A.R., Meneses L., Duarte A.R.C., Paiva A. (2021). Natural deep eutectic systems, an emerging class of cryoprotectant agents. Cryobiology.

[87] Faustino L.R., Silva C.M.G., Rossetto R., Rodrigues G.Q., Figueiredo J.R., Rodrigues A.P.R. (2011). Current status and challenges of cryopreservation of ovarian tissue in mammals. Rev. Bras. De Reprodução Anim..

[88] Picoli T., Barbosa J.S., Vargas G.D., de Oliveira Hübner S., Fischer G. (2015). Toxicidade e eficiência do dimetilsulfóxido (dmso) no congelamento de células madin-darby bovine kidney (mdbk). Sci. Anim. Health.

[89] Bove K.E. (1966). Ethylene glycol toxicity. Am. J. Clin. Pathol..

[90] da Silva G.P., Contiero J., Ávila Neto P.M., de Lima C.J.B. (2014). 1,3-Propanediol: Production, applications and biotechnological potential. Química Nova.

[91] Lewis J.K., Bischof J.C., Braslavsky I., Brockbank K.G.M., Fahy G.M., Fuller B.J., Rabin Y., Tocchio A., Woods E.J., Wowk B.G. (2016). The Grand Challenges of Organ Banking: Proceedings from the first global summit on complex tissue cryopreservation. Cryobiology.

[92] Jesus A.R., Duarte A.R.C., Paiva A. (2022). Use of natural deep eutectic systems as new cryoprotectant agents in the vitrification of mammalian cells. Sci. Rep..

[93] Benoit C., Virginie C., Boris V., Verpoorte R., Witkamp G.J., Choi Y.H. (2021). Chapter Twelve—The use of NADES to support innovation in the cosmetic industry. Advances in Botanical Research.

[94] Dai Y., Rozema E., Verpoorte R., Choi Y.H. (2016). Application of natural deep eutectic solvents to the extraction of anthocyanins from Catharanthus roseus with high extractability and stability replacing conventional organic solvents. J. Chromatogr. A.

[95] Teplova V.V., Isakova E.P., Klein O.I., Dergachova D.I., Gessler N.N., Deryabina Y.I. (2018). Natural Polyphenols: Biological Activity, Pharmacological Potential, Means of Metabolic Engineering (Review). Appl. Biochem. Microbiol..

[96] Panić M., Drakula S., Cravotto G., Verpoorte R., Hruškar M., Radojčić Redovniković I., Radošević K. (2020). Biological activity and sensory evaluation of cocoa by-products NADES extracts used in food fortification. Innov. Food Sci. Emerg. Technol..

[97] Gereniu C.R.N., Saravana P.S., Chun B.S. (2018). Recovery of carrageenan from Solomon Islands red seaweed using ionic liquid-assisted subcritical water extraction. Sep. Purif. Technol..

[98] Ozturk B., Parkinson C., Gonzalez-Miquel M. (2018). Extraction of polyphenolic antioxidants from orange peel waste using deep eutectic solvents. Sep. Purif. Technol..

[99] Ullah H., Wilfred C.D., Shaharun M.S. (2019). Comparative assessment of various extraction approaches for the isolation of essential oil from polygonum minus using ionic liquids. J. King Saud Univ. Sci..

[100] Boussetta N., Lanoisellé J.L., Bedel-Cloutour C., Vorobiev E. (2009). Extraction of soluble matter from grape pomace by high voltage electrical discharges for polyphenol recovery: Effect of sulphur dioxide and thermal treatments. J. Food Eng..

[101] Rockenbach I.I., Rodrigues E., Gonzaga L.V., Caliari V., Genovese M.I., Gonçalves A.E.S.S., Fett R. (2011). Phenolic compounds content and antioxidant activity in pomace from selected red grapes (*Vitis vinifera* L. and *Vitis labrusca* L.) widely produced in Brazil. Food Chem..

[102] Rockenbach I.I., Gonzaga L.V., Rizelio V.M., Gonçalves A.E.d.S.S., Genovese M.I., Fett R. (2011). Phenolic compounds and antioxidant activity of seed and skin extracts of red grape (*Vitis vinifera* and *Vitis labrusca*) pomace from Brazilian winemaking. Food Res. Int..

[103] González-Centeno M.R., Knoerzer K., Sabarez H., Simal S., Rosselló C., Femenia A. (2014). Effect of acoustic frequency and power density on the aqueous ultrasonic-assisted extraction of grape pomace (*Vitis vinifera* L.)—A response surface approach. Ultrason. Sonochemistry.

[104] Li X.L., Cai Y.Q., Qin H., Wu Y.J. (2008). Therapeutic effect and mechanism of proanthocyanidins from grape seeds in rats with TNBS-induced ulcerative colitis. Can. J. Physiol. Pharmacol..

[105] Cheah K.Y., Bastian S.E., Acott T.M., Abimosleh S.M., Lymn K.A., Howarth G.S. (2013). Grape seed extract reduces the severity of selected disease markers in the proximal colon of dextran sulphate sodium-induced colitis in rats. Dig. Dis. Sci..

[106] Wang Y.H., Yang X.L., Wang L., Cui M.X., Cai Y.Q., Li X.L., Wu Y.J. (2010). Effects of proanthocyanidins from grape seed on treatment of recurrent ulcerative colitis in rats. Can. J. Physiol. Pharmacol..

[107] Apostolou A., Stagos D., Galitsiou E., Spyrou A., Haroutounian S., Portesis N., Trizoglou I., Hayes A.W., Tsatsakis A.M., Kouretas D. (2013). Assessment of polyphenolic content, antioxidant activity, protection against ROS-induced DNA damage and anticancer activity of Vitis vinifera stem extracts. Food Chem. Toxicol..

[108] Teixeira A., Baenas N., Dominguez-Perles R., Barros A., Rosa E., Moreno D.A., Garcia-Viguera C. (2014). Natural Bioactive Compounds from Winery By-Products as Health Promoters: A Review. Int. J. Mol. Sci..

[109] Vislocky L.M., Fernandez M.L. (2010). Biomedical effects of grape products. Nutr. Rev..

[110] Xia E.Q., Deng G.F., Guo Y.J., Li H.B. (2010). Biological Activities of Polyphenols from Grapes. Int. J. Mol. Sci..

[111] Hayyan M., Hashim M.A., Hayyan A., Al-Saadi M.A., AlNashef I.M., Mirghani M.E.S., Saheed O.K. (2013). Are deep eutectic solvents benign or toxic?. Chemosphere.

[112] Bajkacz S., Adamek J. (2017). Evaluation of new natural deep eutectic solvents for the extraction of isoflavones from soy products. Talanta.

[113] Munro I.C., Harwood M., Hlywka J.J., Stephen A.M., Doull J., Flamm W.G., Adlercreutz H. (2003). Soy Isoflavones: A Safety Review. Nutr. Rev..

[114] Sarkar F.H., Li Y. (2003). Soy Isoflavones and Cancer Prevention. Cancer Investig..

[115] Meng Z., Zhao J., Duan H., Guan Y., Zhao L. (2018). Green and efficient extraction of four bioactive flavonoids from Pollen Typhae by ultrasound-assisted deep eutectic solvents extraction. J. Pharm. Biomed. Anal..

[116] Sang J., Li B., Huang Y., Ma Q., Liu K., Li C.Q. (2018). Deep eutectic solvent-based extraction coupled with green two-dimensional HPLC-DAD-ESI-MS/MS for the determination of anthocyanins from *Lycium ruthenicum* Murr. fruit. Anal. Methods.

[117] García A., Rodríguez-Juan E., Rodríguez-Gutiérrez G., Rios J.J., Fernández-Bolaños J. (2016). Extraction of phenolic compounds from virgin olive oil by deep eutectic solvents (DESs). Food Chem..

[118] Bonacci S., Di Gioia M.L., Costanzo P., Maiuolo L., Tallarico S., Nardi M. (2020). Natural Deep Eutectic Solvent as Extraction Media for the Main Phenolic Compounds from Olive Oil Processing Wastes. Antioxidants.

[119] Beauchamp G.K., Keast R.S.J., Morel D., Lin J., Pika J., Han Q., Lee C.H., Smith A.B., Breslin P.A.S. (2005). Ibuprofen-like activity in extra-virgin olive oil. Nature.

[120] Lozano-Castellón J., López-Yerena A., Rinaldi de Alvarenga J.F., Romero del Castillo-Alba J., Vallverdú-Queralt A., Escribano-Ferrer E., Lamuela-Raventós R.M. (2020). Health-promoting properties of oleocanthal and oleacein: Two secoiridoids from extra-virgin olive oil. Crit. Rev. Food Sci. Nutr..

[121] Maheswari V., Babu P.A.S. (2022). Phlorotannin and its derivatives, a potential antiviral molecule from brown seaweeds, an overview. Russ. J. Mar. Biol..

[122] Pradhan B., Nayak R., Bhuyan P.P., Patra S., Behera C., Sahoo S., Ki J.S., Quarta A., Ragusa A., Jena M. (2022). Algal phlorotannins as novel antibacterial agents with reference to the antioxidant modulation: Current advances and future directions. Mar. Drugs.

[123] Wang T., Jónsdóttir R., Liu H., Gu L., Kristinsson H.G., Raghavan S., Ólafsdóttir G. (2012). Antioxidant capacities of phlorotannins extracted from the brown algae *Fucus vesiculosus*. J. Agric. Food Chem..

[124] Wang C., Li X., Jin L., Zhao Y., Zhu G., Shen W. (2019). Dieckol inhibits non-small–cell lung cancer cell proliferation and migration by regulating the PI3K/AKT signaling pathway. J. Biochem. Mol. Toxicol..

[125] Kim A.R., Shin T.S., Lee M.S., Park J.Y., Park K.E., Yoon N.Y., Kim J.S., Choi J.S., Jang B.C., Byun D.S. (2009). Isolation and identification of phlorotannins from *Ecklonia stolonifera* with antioxidant and anti-inflammatory properties. J. Agric. Food Chem..

[126] Cui Y., Amarsanaa K., Lee J.H., Rhim J.K., Kwon J.M., Kim S.H., Park J.M., Jung S.C., Eun S.Y. (2019). Neuroprotective mechanisms of dieckol against glutamate toxicity through reactive oxygen species scavenging and nuclear factor-like 2/heme oxygenase-1 pathway. Korean J. Physiol. Pharmacol..

[127] Wang L., Je J.G., Yang H.W., Jeon Y.J., Lee S. (2021). Dieckol, an algae-derived phenolic compound, suppresses UVB-induced skin damage in human dermal fibroblasts and its underlying mechanisms. Antioxidants.

[128] Catarino M.D., Pires S.M., Silva S., Costa F., Braga S.S., Pinto D.C., Silva A.M.S., Cardoso S.M. (2022). Overview of phlorotannins’ constituents in Fucales. Mar. Drugs.

[129] Zayed A., Ulber R. (2020). Fucoidans: Downstream processes and recent applications. Mar. Drugs.

[130] Zheng H., Zhao Y., Guo L. (2022). A bioactive substance derived from brown seaweeds: Phlorotannins. Mar. Drugs.

[131] Obluchinskaya E.D., Pozharitskaya O.N., Zakharova L.V., Daurtseva A.V., Flisyuk E.V., Shikov A.N. (2021). Efficacy of natural deep eutectic solvents for extraction of hydrophilic and lipophilic compounds from *Fucus vesiculosus*. Molecules.

[132] Mustafa N.R., Spelbos V.S., Witkamp G.J., Verpoorte R., Choi Y.H. (2021). Solubility and stability of some pharmaceuticals in natural deep eutectic solvents-based formulations. Molecules.

[133] Jabeur I., Pereira E., Barros L., Calhelha R.C., Soković M., Oliveira M.B.P.P., Ferreira I.C.F.R. (2017). *Hibiscus sabdariffa* L. as a source of nutrients, bioactive compounds and colouring agents. Food Res. Int..

[134] Alañón M.E., Ivanović M., Pimentel-Mora S., Borrás-Linares I., Arráez-Román D., Segura-Carretero A. (2020). A novel sustainable approach for the extraction of value-added compounds from *Hibiscus sabdariffa* L. calyces by natural deep eutectic solvents. Food Res. Int..

[135] Fu J.J., Sun C., Xu X.B., Zhou D.Y., Song L., Zhu B.W. (2020). Improving the functional properties of bovine serum albumin-glucose conjugates in natural deep eutectic solvents. Food Chem..

[136] Velásquez P., Bustos D., Montenegro G., Giordano A. (2021). Ultrasound-Assisted Extraction of Anthocyanins Using Natural Deep Eutectic Solvents and Their Incorporation in Edible Films. Molecules.

[137] Chandra Roy V., Ho T.C., Lee H.J., Park J.S., Nam S.Y., Lee H., Getachew A.T., Chun B.S. (2021). Extraction of astaxanthin using ultrasound-assisted natural deep eutectic solvents from shrimp wastes and its application in bioactive films. J. Clean. Prod..

[138] Chattopadhyay S., Raines R.T. (2014). Collagen-based biomaterials for wound healing. Biopolymers.

[139] Grønlien K.G., Pedersen M.E., Tønnesen H.H. (2020). A natural deep eutectic solvent (NADES) as potential excipient in collagen-based products. Int. J. Biol. Macromol..

[140] Fang X., Li Y., Kua Y.L., Chew Z.L., Gan S., Tan K.W., Lee T.Z.E., Cheng W.K., Lau H.L.N. (2022). Insights on the potential of natural deep eutectic solvents (NADES) to fine-tune durian seed gum for use as edible food coating. Food Hydrocoll..

[141] Tian Y., Sun D.W., Zhu Z. (2022). Development of natural deep eutectic solvents (NADESs) as anti-freezing agents for the frozen food industry: Water-tailoring effects, anti-freezing mechanisms and applications. Food Chem..

[142] da Silva D.T., Rodrigues R.F., Machado N.M., Maurer L.H., Ferreira L.F., Somacal S., da Veiga M.L., Vizzoto M., Rodrigues E., Barcia M.T. (2020). Natural deep eutectic solvent (NADES)-based blueberry extracts protect against ethanol-induced gastric ulcer in rats. Food Res. Int..

[143] Grozdanova T., Trusheva B., Alipieva K., Popova M., Dimitrova L., Najdenski H., Zaharieva M.M., Ilieva Y., Vasileva B., Miloshev G. (2020). Extracts of medicinal plants with natural deep eutectic solvents: Enhanced antimicrobial activity and low genotoxicity. BMC Chem..

[144] Doldolova K., Bener M., Lalikoğlu M., Aşçı Y.S., Arat R., Apak R. (2021). Optimization and modeling of microwave-assisted extraction of curcumin and antioxidant compounds from turmeric by using natural deep eutectic solvents. Food Chem..

[145] Bener M., Özyürek M., Güçlü K., Apak R. (2016). Optimization of Microwave-Assisted Extraction of Curcumin from *Curcuma longa* L. (Turmeric) and Evaluation of Antioxidant Activity in Multi-Test Systems. Rec. Nat. Prod..

[146] Farhood B., Mortezaee K., Goradel N.H., Khanlarkhani N., Salehi E., Nashtaei M.S., Najafi M., Sahebkar A. (2019). Curcumin as an anti-inflammatory agent: Implications to radiotherapy and chemotherapy. J. Cell. Physiol..

[147] Naeini M.B., Momtazi A.A., Jaafari M.R., Johnston T.P., Barreto G., Banach M., Sahebkar A. (2019). Antitumor effects of curcumin: A lipid perspective. J. Cell. Physiol..

[148] Panossian A., Wikman G., Sarris J. (2010). Rosenroot (*Rhodiola rosea*): Traditional use, chemical composition, pharmacology and clinical efficacy. Phytomedicine.

[149] Galambosi B., Galambosi Z., Hethelyi E., Szöke E., Volodin V., Poletaeva I., Iljina I. (2010). Importance and quality of roseroot (*Rhodiola rosea* L.) growing in the European North. Z. Für Arznei-Gewürzpflanzen.

[150] Brown R.P., Gerbarg P.L., Ramazanov Z. (2002). *Rhodiola rosea*. Phytomedicinal Overv. HerbalGram.

[151] Khanum F., Bawa A.S., Singh B. (2005). *Rhodiola rosea*: A versatile adaptogen. Compr. Rev. Food Sci. Food Saf..

[152] Rohloff J. (2002). Volatiles from rhizomes of *Rhodiola rosea* L.. Phytochemistry.

[153] Ioset K.N., Nyberg N.T., Van Diermen D., Malnoe P., Hostettmann K., Shikov A.N., Jaroszewski J.W. (2011). Metabolic profiling of *Rhodiola rosea* rhizomes by 1H NMR spectroscopy. Phytochem. Anal..

[154] Tao H., Wu X., Cao J., Peng Y., Wang A., Pei J., Xiao J., Wang S., Wang Y. (2019). Rhodiola species: A comprehensive review of traditional use, phytochemistry, pharmacology, toxicity, and clinical study. Med. Res. Rev..

[155] Shikov A.N., Kosman V.M., Flissyuk E.V., Smekhova I.E., Elameen A., Pozharitskaya O.N. (2020). Natural deep eutectic solvents for the extraction of phenyletanes and phenylpropanoids of *Rhodiola rosea* L.. Molecules.

[156] Wahab S., Annadurai S., Abullais S.S., Das G., Ahmad W., Ahmad M.F., Kandasamy G., Vasudevan R., Ali M.S., Amir M. (2021). *Glycyrrhiza glabra* (Licorice): A comprehensive review on its phytochemistry, biological activities, clinical evidence and toxicology. Plants.

[157] Cheng M., Zhang J., Yang L., Shen S., Li P., Yao S., Qu H., Li J., Yao C., Wei W. (2021). Recent advances in chemical analysis of licorice (Gan-Cao). Fitoterapia.

[158] Gravel I. (2005). The content of trace elements in biologically active additives and their aqueous extracts. Farmatsiia-Moskva.

[159] Gravel I., Tsoi Y., Denisova T., Khotimchenko S. (2010). Heavy metals in the raw materials and infusions of peppermint (*Mentha piperita*). Pharm. (Farmatsiya).

[160] Tsvetov N., Drogobuzhskaya S. (2021). Recovery of Some Elements from Empetrum nigrum L. Growing in the Kola Peninsula Using Acid-Based Deep Eutectic Solvents.

[161] Shikov A.N., Obluchinskaya E.D., Flisyuk E.V., Terninko I.I., Generalova Y.E., Pozharitskaya O.N. (2022). The impact of natural deep eutectic solvents and extraction method on the co-extraction of trace metals from *Fucus vesiculosus*. Mar. Drugs.

[162] Shikov A.N., Shikova V.A., Whaley A.O., Burakova M.A., Flisyuk E.V., Whaley A.K., Terninko I.I., Generalova Y.E., Gravel I.V., Pozharitskaya O.N. (2022). The ability of acid-based natural deep eutectic solvents to co-extract elements from the roots of *Glycyrrhiza glabra* L. and associated health risks. Molecules.

