# Supplementary material for: From Nature to Innovation: The Uncharted Potential of Natural Deep Eutectic Solvents
Source: Molecules. 2023 Nov 18;28(22):7653. doi: 10.3390/molecules28227653 (PMC10675409; doi:10.3390/molecules28227653)
Supplement: Supplementary file 1 [file molecules-28-07653-s001.zip › molecules-2675962-supplementary-revised.pdf]

Tables S1a and S1b are for articles published in journals with the highest impact factors (for Web of Science) and the highest CiteScore (for Scopus).

S1a. Most Relevant Journals (Web of Science)

WoS			
**Authors**	**Article Name**	**Journal**	**2022 JIF**
Satlewal, A; Agrawal, R; Bhagia, S; Sangoro, J; Ragauskas, AJ	Natural deep eutectic solvents for lignocellulosic biomass pretreatment: Recent developments, challenges and novel opportunities	BIOTECHNOLOGY ADVANCES	16
El Achkar, T; Greige-Gerges, H; Fourmentin, S	Basics and properties of deep eutectic solvents: a review	ENVIRONMENTAL CHEMISTRY LETTERS	15.7
Benvenutti, L; Zielinski, AAF; Ferreira, SRS	Which is the best food emerging solvent: IL, DES or NADES?	TRENDS IN FOOD SCIENCE & TECHNOLOGY	15.3
Tian, Y; Zhu, ZW; Sun, DW	Naturally sourced biosubstances for regulating freezing points in food researches: Fundamentals, current applications and future trends	TRENDS IN FOOD SCIENCE & TECHNOLOGY	15.3
Yu, JH; Liu, XW; Zhang, LF; Shao, P; Wu, WN; Chen, ZR; Li, JD; Renard, CMGC	An overview of carotenoid extractions using green solvents assisted by Z-isomerization	TRENDS IN FOOD SCIENCE & TECHNOLOGY	15.3
Cui, HP; Yu, JH; Zhai, Y; Feng, LH; Chen, PS; Hayat, K; Xu, Y; Zhang, XM; Ho, CT	Formation and fate of Amadori rearrangement products in Maillard reaction	TRENDS IN FOOD SCIENCE & TECHNOLOGY	15.3
Gullon, P; Gullon, B; Romani, A; Rocchetti, G; Lorenzo, JM	Smart advanced solvents for bioactive compounds recovery from agri-food by-products: A review	TRENDS IN FOOD SCIENCE & TECHNOLOGY	15.3
Liu, XP; Zhai, YB; Xu, ZX; Zhu, Y; Zhou, Y; Wang, ZX; Liu, LM; Liang, FS; Ren, WY; Xie, Y; Li, CT; Xu, M	One-pot production of 5-methylfurfural (5-MF) and enhanced dewaterability of waste activated sludge by hydrothermal treatment with natural deep eutectic solvents (NADES): Experimental and theoretical studies	CHEMICAL ENGINEERING JOURNAL	15.1

Cai, ZH; Wang, JD; Liu, L; Ruan, LD; Gu, Q; Yan, XY; Fu, LN; Zhao, PQ; Zhang, S; Fu, YJ	A green and designable natural deep eutectic solvent-based supramolecular solvents system: Efficient extraction and enrichment for phytochemicals	CHEMICAL ENGINEERING JOURNAL	15.1
Moradi, G; Rahimi, M; Zinadini, S; Shamsipur, M; Babajani, N	Natural deep eutectic solvent modified nanofiltration membranes with superior antifouling properties for pharmaceutical wastewater treatment	CHEMICAL ENGINEERING JOURNAL	15.1
Zhang, P; Xiong, WJ; Shi, MZ; Tu, ZH; Hu, XB; Zhang, XM; Wu, YT	Natural deep eutectic solvent-based gels with multi-site interaction mechanism for selective membrane separation of SO2 from N2 and CO2	CHEMICAL ENGINEERING JOURNAL	15.1
Xu, L; Sun, DW; Tian, Y; Fan, TH; Zhu, ZW	Nanocomposite hydrogel for daytime passive cooling enabled by combined effects of radiative and evaporative cooling	CHEMICAL ENGINEERING JOURNAL	15.1
Du, X; Wang, B; Li, HJ; Liu, HT; Shi, S; Feng, J; Pan, N; Xia, XF	Research progress on quality deterioration mechanism and control technology of frozen muscle foods	COMPREHENSIVE REVIEWS IN FOOD SCIENCE AND FOOD SAFETY	14.8
Candia-Lomeli, M; Covarrubias-Garcia, I; Aizpuru, A; Arriaga, S	Preparation and physicochemical characterization of deep eutectic solvents and ionic liquids for the potential absorption and biodegradation of styrene vapors	JOURNAL OF HAZARDOUS MATERIALS	13.6
Espino, M; Fernandez, MD; Gomez, FJV; Silva, MF	Natural designer solvents for greening analytical chemistry	TRAC-TRENDS IN ANALYTICAL CHEMISTRY	13.1
Cunha, SC; Fernandes, JO	Extraction techniques with deep eutectic solvents	TRAC-TRENDS IN ANALYTICAL CHEMISTRY	13.1
Della Posta, S; Gallo, V; Gentili, A; Fanali, C	Strategies for the recovery of bioactive molecules from deep eutectic solvents extracts	TRAC-TRENDS IN ANALYTICAL CHEMISTRY	13.1
Wang, YL; Zhang, YB; Lin, ZS; Huang, T; Li, W; Gong, WX; Guo, YH; Su, JM; Wang, JY; Tu, Q	A green method of preparing a natural and degradable wound dressing containing aloe vera as an active ingredient	COMPOSITES PART B-ENGINEERING	13.1
Lavilla, I; Romero, V; Costas, I; Bendicho, C	Greener derivatization in analytical chemistry	TRAC-TRENDS IN ANALYTICAL CHEMISTRY	13.1

Hashemi, B; Zohrabi, P; Dehdashtian, S	Application of green solvents as sorbent modifiers in sorptive-based extraction techniques for extraction of environmental pollutants	TRAC-TRENDS IN ANALYTICAL CHEMISTRY	13.1
Hasani, M; Kalhor, HR	Enzyme-Inspired Lysine-Modified Carbon Quantum Dots Performing Carbonylation Using Urea and a Cascade Reaction for Synthesizing 2-Benzoxazolinone	ACS CATALYSIS	12.9

S1b. Most Relevant Journals (Scopus)

Scopus			
**Authors**	**Article Name**	**Journal**	**2022 CS**
Cunha S.C., Fernandes J.O.,	Extraction techniques with deep eutectic solvents	TrAC - Trends in Analytical Chemistry	25.8
Espino M., de los Ángeles Fernández M., Gomez F.J.V., Silva M.F.,	Natural designer solvents for greening analytical chemistry	TrAC - Trends in Analytical Chemistry	25.8
Tian Y., Zhu Z., Sun D.-W.,	Naturally sourced biosubstances for regulating freezing points in food researches: Fundamentals, current applications and future trends	Trends in Food Science and Technology	25.2
Benvenutti L., Zielinski A.A.F., Ferreira S.R.S.,	Which is the best food emerging solvent: IL, DES or NADES?	Trends in Food Science and Technology	25.2
Mišan A., Nađpal J., Stupar A., Pojić M., Mandić A., Verpoorte R., Choi Y.H.,	The perspectives of natural deep eutectic solvents in agri-food sector	Critical Reviews in Food Science and Nutrition	23.6
Liu X., Zhai Y., Xu Z., Zhu Y., Zhou Y., Wang Z., Liu L., Liang F., Ren W., Xie Y., Li C., Xu M.,	One-pot production of 5-methylfurfural (5-MF) and enhanced dewaterability of waste activated sludge by hydrothermal treatment with natural deep eutectic solvents (NADES): Experimental and theoretical studies	Chemical Engineering Journal	21.5

Cai Z.-H., Wang J.-D., Liu L., Ruan L.-D., Gu Q., Yan X.-Y., Fu L.-N., Zhao P.-Q., Zhang S., Fu Y.-J.,	A green and designable natural deep eutectic solvent-based supramolecular solvents system: Efficient extraction and enrichment for phytochemicals	Chemical Engineering Journal	21.5
Hasani M., Kalhor H.R.,	Enzyme-Inspired Lysine-Modified Carbon Quantum Dots Performing Carbonylation Using Urea and a Cascade Reaction for Synthesizing 2-Benzoxazolinone	ACS Catalysis	20.6
Candia-Lomelí M., Covarrubias-Garcia I., Aizpuru A., Arriaga S.,	Preparation and physicochemical characterization of deep eutectic solvents and ionic liquids for the potential absorption and biodegradation of styrene vapors	Journal of Hazardous Materials	20.2
Nava-Ocampo M.F., Fuhaid L.A., Verpoorte R., Choi Y.H., van Loosdrecht M.C.M., Vrouwenvelder J.S., Witkamp G.J., Farinha A.S.F., Bucs S.S.,	Natural deep eutectic solvents as biofilm structural breakers	Water Research	19.8
Zhong C., Luo S., Ye J., Liu C.,	Shape and size-controlled starch nanoparticles prepared by self-assembly in natural deep eutectic solvents: Effect and mechanism	Food Hydrocolloids	19.3
Fang X., Li Y., Kua Y.L., Chew Z.L., Gan S., Tan K.W., Lee T.Z.E., Cheng W.K., Lau H.L.N.,	Insights on the potential of natural deep eutectic solvents (NADES) to fine-tune durian seed gum for use as edible food coating	Food Hydrocolloids	19.3
Grala D., Biernacki K., Freire C., Kuźniarska-Biernacka I., Souza H.K.S., Gonçalves M.P.,	Effect of natural deep eutectic solvent and chitosan nanoparticles on physicochemical properties of locust bean gum films	Food Hydrocolloids	19.3
Zheng W.-Y., Wu X.-M., Li M.-X., Qiu S.-L., Yang T.-D., Yang R., Chen Z.-P., Wang S.-Y., Liao L.,	Synergistic strongly coupled super-deamidation of wheat gluten by glucose-organic acid natural deep eutectic solvent and the efficaciousness of structure and functionality	Food Hydrocolloids	19.3
Chen M., Lahaye M.,	Natural deep eutectic solvents pretreatment as an aid for pectin extraction from apple pomace	Food Hydrocolloids	19.3
van der Sman R.G.M., van den Hoek I.A.F., Renzetti S.,	Sugar replacement with zwitterionic plasticizers like amino acids	Food Hydrocolloids	19.3

Gouveia T.I.A., Biernacki K., Castro M.C.R., Gonçalves M.P., Souza H.K.S.,	A new approach to develop biodegradable films based on thermoplastic pectin	Food Hydrocolloids	19.3
Liu C., Li Z., Li M.-C., Chen W., Xu W., Hong S., Wu Q., Mei C.,	Lignin-containing cellulose nanofibers made with microwave-aid green solvent treatment for magnetic fluid stabilization	Carbohydrate Polymers	18.9
Chen M., Falourd X., Lahaye M.,	Sequential natural deep eutectic solvent pretreatments of apple pomace: A novel way to promote water extraction of pectin and to tailor its main structural domains	Carbohydrate Polymers	18.9
Douard L., Bras J., Encinas T., Belgacem M.N.,	Natural acidic deep eutectic solvent to obtain cellulose nanocrystals using the design of experience approach	Carbohydrate Polymers	18.9
Selvanathan V., Azzahari A.D., Abd. Halim A.A., Yahya R.,	Ternary natural deep eutectic solvent (NADES) infused phthaloyl starch as cost efficient quasi-solid gel polymer electrolyte	Carbohydrate Polymers	18.9

Table S2. The Top 20 Publishers

**S2. The Top 20 Publishers**					
**WoS**			**Scopus**		
**Publication Titles**	**Total**	**% of 1036**	**Publication Titles**	**Total**	**% of 1055**
JOURNAL OF MOLECULAR LIQUIDS	73	7,05%	JOURNAL OF MOLECULAR LIQUIDS	78	7.39%
MOLECULES	54	5,21%	MOLECULES	54	5.12%
FOOD CHEMISTRY	35	3,38%	FOOD CHEMISTRY	37	3.51%
ACS SUSTAINABLE CHEMISTRY ENGINEERING	34	3,28%	ACS SUSTAINABLE CHEMISTRY ENGINEERING	33	3.13%
SEPARATION AND PURIFICATION TECHNOLOGY	19	1,83%	INDUSTRIAL CROPS AND PRODUCTS	21	1.99%
ANTIOXIDANTS	18	1,74%	SEPARATION AND PURIFICATION TECHNOLOGY	20	1.90%
JOURNAL OF CHROMATOGRAPHY A	17	1,64%	MICROCHEMICAL JOURNAL	18	1.71%
INDUSTRIAL CROPS AND PRODUCTS	16	1,54%	ANTIOXIDANTS	17	1.61%

MICROCHEMICAL JOURNAL	16	1,54%	JOURNAL OF CHROMATOGRAPHY A	17	1.61%
RSC ADVANCES	16	1,54%	RSC ADVANCES	16	1.52%
GREEN CHEMISTRY	15	1,45%	SUSTAINABLE CHEMISTRY AND PHARMACY	16	1.52%
PLANTA MEDICA	14	1,35%	GREEN CHEMISTRY	15	1.42%
PLANTS BASEL	14	1,35%	PLANTS	14	1.33%
ADVANCES IN BOTANICAL RESEARCH	13	1,25%	ADVANCES IN BOTANICAL RESEARCH	13	1.23%
EUTECTIC SOLVENTS AND STRESS IN PLANTS	13	1,25%	FOODS	12	1.14%
SUSTAINABLE CHEMISTRY AND PHARMACY	13	1,25%	INTERNATIONAL JOURNAL OF BIOLOGICAL MACROMOLECULES	12	1.14%
FOODS	12	1,16%	JOURNAL OF AGRICULTURAL AND FOOD CHEMISTRY	10	0.95%
INTERNATIONAL JOURNAL OF BIOLOGICAL MACROMOLECULES	11	1,06%	LWT	10	0.95%
JOURNAL OF AGRICULTURAL AND FOOD CHEMISTRY	11	1,06%	TALANTA	10	0.95%
TALANTA	10	0,97%	CARBOHYDRATE POLYMERS	9	0.85%

OBS.: The review conducted by the TEMAC method continues to evaluate the journals that publish the most on the subject, this can guide the path of the researcher when directing his article to be published in the journals that are already publishing on and to know more deeply about the topic addressed.

Table S3. The Top 20 Authors

**S3. The Top 20 Authors**					
**WoS**			**Scopus**		
**Author Name**	**Total**	**% of 1036**	**Author Name**	**Total**	**% of 1055**
Duarte ARC	27	2,61%	Duarte ARC	27	2.56%
Paiva A	25	2,41%	Verpoorte R	27	2.56%
Silva MF	25	2,41%	Silva MF	25	2.37%
Verpoorte R	25	2,41%	Choi YH	24	2.27%
Choi YH	24	2,32%	Paiva A	24	2.27%
Khan MA	24	2,32%	Atilhan M	19	1.80%

Gomez FJV	18	1,74%	Gomez FJV	19	1.80%
Atilhan M	17	1,64%	Aparicio S	15	1.42%
Redovnikovic IR	17	1,64%	Reis RL	14	1.33%
Aparicio S	16	1,54%	Espino M	13	1.23%
Espino M	13	1,25%	Radojčić Redovniković I	13	1.23%
Panic M	13	1,25%	Dai Y	11	1.04%
Fernandez MD	12	1,16%	Panić M	11	1.04%
Mun'im A	12	1,16%	Tiecco M	11	1.04%
Bubalo MC	11	1,06%	Boudesocque-Delaye L	10	0.95%
Grasemann H	11	1,06%	Witkamp GJ	10	0.95%
Reis RL	11	1,06%	Altunay N	9	0.85%
Tiecco M	11	1,06%	Craveiro R	9	0.85%
Boudesocque-delaye L	10	0,97%	Cvjetko Bubalo M	9	0.85%
Dai YT	10	0,97%	Mun′im A	9	0.85%
Douda DN	10	0,97%	Wils L	9	0.85%
Liu Y	10	0,97%	Zhao L	9	0.85%
Liu YF	10	0,97%	Ahmad I	8	0.76%
Witkamp GJ	10	0,97%	Boczkaj G	8	0.76%
Zhao LS	10	0,97%	Bravi M	8	0.76%

OBS.: Knowing the authors who publish the most on the subject and the articles they are publishing is another analysis conducted by TEMAC. The pattern of positions remains constant in all analyzes carried out by both Web of Science and Scopus.

Table S4. The Top 20 Cited Authors

**S4. The Top 20 Cited Authors**			
**WoS**		**Scopus**	
**Author Name**	**Cited Times**	**Author Name**	**Cited Times**
Choi YH	4151	Verpoorte R	4680
Verpoorte R	4138	Choi YH	4678
Dai Y	3798	Dai Y	4280

Witkamp GJ	3159	Witkamp G-J	3500
vanSpronsen J	2318	vanSpronsen J	2566
Duarte ARC	2284	Paiva A	2172
Paiva A	1797	Duarte ARC	2146
Reis RL	1773	Reis RL	2111
Craveiro R	1696	Craveiro R	1819
Martins M	1429	Aroso I	1657
Aroso I	1322	Martins M	1455
Khan MA	1295	Radojčić Redovniković I	966
Grasemann H	1200	Hollmann F	869
Radojčić Redovniković I	868	Silva MF	866
Hollmann F	790	Liu Y	858
Espino M	768	Espino M	799
Arends IWCE	701	Gomez FJV	785
Verberne M	680	Verberne M	772
Silva MF	680	Arends IWCE	772
de los Angeles Fernandez M	641	Cvjetko Bubalo M	717

OBS.: Knowing which authors are most cited differs from those who publish the most. Citation analysis is done using all the results of the articles obtained, individualizing each of the authors, and adding the history of total citations received for them contained in the database platforms.

Table S5. Evolution of the Theme

**S5. Evolution of the Theme**			
**WoS**		**Scopus**	
**Year**	**Number of Articles**	**Year**	**Number of Articles**
2022	265	2022	286
2021	208	2021	196
2020	146	2023	169
2019	111	2020	150

2023	110	2019	100
2018	83	2018	77
2017	32	2017	28
2016	29	2016	24
2014	16	2015	10
2015	16	2014	7
2011	8	2013	4
2013	8	2012	0
2012	4	2011	4

OBS.: Monitoring the evolution of articles published on the subject year-by-year is important to pay attention to the research being conducted in the area and whether the research is stagnant or has the potential to cover the area.

Tables S6a and S6b Most Cited Documents

**S6a - Most Cited Documents (Web of Science)**		
	**WoS**	
**Authors**	**Document Title**	**Total Citations**
Dai, Yuntao; van Spronsen, Jaap; Witkamp, Geert-Jan; Verpoorte, Robert; Choi, Young Hae	Natural deep eutectic solvents as new potential media for green technology	1401
Paiva, Alexandre; Craveiro, Rita; Aroso, Ivo; Martins, Marta; Reis, Rui L.; Duarte, Ana Rita C.	Natural Deep Eutectic Solvents - Solvents for the 21st Century	1296
Choi, Young Hae; van Spronsen, Jaap; Dai, Yuntao; Verberne, Marianne; Hollmann, Frank; Arends, Isabel W. C. E.; Witkamp, Geert-Jan; Verpoorte, Robert	Are Natural Deep Eutectic Solvents the Missing Link in Understanding Cellular Metabolism and Physiology?	680
Dai, Yuntao; Witkamp, Geert-Jan; Verpoorte, Robert; Choi, Young Hae	Tailoring properties of natural deep eutectic solvents with water to facilitate their applications	622

Liu, Yang; Friesen, J. Brent; McAlpine, James B.; Lankin, David C.; Chen, Shao-Nong; Pauli, Guido F.	Natural Deep Eutectic Solvents: Properties, Applications, and Perspectives	507
Dai, Yuntao; Witkamp, Geert-Jan; Verpoorte, Robert; Choi, Young Hae	Natural Deep Eutectic Solvents as a New Extraction Media for Phenolic Metabolites in Carthamus tinctorius L.	427
Ruesgas-Ramon, Mariana; Figueroa-Espinoza, Maria Cruz; Durand, Erwann	Application of Deep Eutectic Solvents (DES) for Phenolic Compounds Extraction: Overview, Challenges, and Opportunities	368
Cunha, Sara C.; Fernandes, Jose O.	Extraction techniques with deep eutectic solvents	343
van Osch, Dannie J. G. P.; Dietz, Carin H. J. T.; van Spronsen, Jaap; Kroon, Maaike C.; Gallucci, Fausto; Annaland, Martin van Sint; Tuinier, Remco	A Search for Natural Hydrophobic Deep Eutectic Solvents Based on Natural Components	238
Espino, Magdalena; de los Angeles Fernandez, Maria; Gomez, Federico J. V.; Fernanda Silva, Maria	Natural designer solvents for greening analytical chemistry	226
Dai, Yuntao; Verpoorte, Robert; Choi, Young Hae	Natural deep eutectic solvents providing enhanced stability of natural colorants from safflower (Carthamus tinctorius)	223
Satlewal, Alok; Agrawal, Ruchi; Bhagia, Samarthya; Sangoro, Joshua; Ragauskas, Arthur J.	Natural deep eutectic solvents for lignocellulosic biomass pretreatment: Recent developments, challenges and novel opportunities	218

Dai, Yuntao; Rozema, Evelien; Verpoorte, Robert; Choi, Young Hae	Application of natural deep eutectic solvents to the extraction of anthocyanins from Catharanthus roseus with high extractability and stability replacing conventional organic solvents	215
Craveiro, R.; Aroso, I.; Flammia, V.; Carvalho, T.; Viciosa, M. T.; Dionisio, M.; Barreiros, S.; Reis, R. L.; Duarte, A. R. C.; Paiva, A.	Properties and thermal behavior of natural deep eutectic solvents	208
Huang, Yao; Feng, Fang; Jiang, Jie; Qiao, Ying; Wu, Tao; Voglmeir, Josef; Chen, Zhi-Gang	Green and efficient extraction of rutin from tartary buckwheat hull by using natural deep eutectic solvents	204
Vanda, Henni; Dai, Yuntao; Wilson, Erica G.; Verpoorte, Robert; Choi, Young Hae	Green solvents from ionic liquids and deep eutectic solvents to natural deep eutectic solvents	199
Bubalo, Marina Cvjetko; Vidovic, Senka; Redovnikovic, Ivana Radojcic; Jokic, Stela	New perspective in extraction of plant biologically active compounds by green solvents	198
Radosevic, Kristina; Curko, Natka; Srcek, Visnja Gaurina; Bubalo, Marina Cvjetko; Tomasevic, Marina; Ganic, Karin Kovacevic; Redovnikovic, Ivana Radojcic	Natural deep eutectic solvents as beneficial extractants for enhancement of plant extracts bioactivity	195
Chemat, Farid; Vian, Maryline Abert; Ravi, Harish Karthikeyan; Khadhraoui, Boutheina; Hilali, Soukaina; Perino, Sandrine; Tixier, Anne-Sylvie Fabiano	Review of Alternative Solvents for Green Extraction of Food and Natural Products: Panorama, Principles, Applications and Prospects	182
Bosiljkov, Tomislau; Dujmic, Filip; Bubalo, Marina Cujetko; Hribar, Janez; Vidrih, Rajko; Brncic, Mladen; Zlatic, Emil; Redovnikavic, Ivana Radojclc; Jokic, Stela	Natural deep eutectic solvents and ultrasound-assisted extraction: Green approaches for extraction of wine lees anthocyanins	178

S6b - Most Cited Documents (Scopus)

Scopus		
**Authors**	**Document Title**	**Total Citations**
Dai Y., van Spronsen J., Witkamp G.-J., Verpoorte R., Choi Y.H.	Natural deep eutectic solvents as new potential media for green technology	1540
Paiva A., Craveiro R., Aroso I., Martins M., Reis R.L., Duarte A.R.C.	Natural deep eutectic solvents - Solvents for the 21st century	1427
Choi Y.H., van Spronsen J., Dai Y., Verberne M., Hollmann F., Arends I.W.C.E., Witkamp G.-J., Verpoorte R.	Are natural deep eutectic solvents the missing link in understanding cellular metabolism and physiology?	772
Dai Y., Witkamp G.-J., Verpoorte R., Choi Y.H.	Tailoring properties of natural deep eutectic solvents with water to facilitate their applications	676
Liu Y., Friesen J.B., McAlpine J.B., Lankin D.C., Chen S.-N., Pauli G.F.	Natural Deep Eutectic Solvents: Properties, Applications, and Perspectives	549
Dai Y., Witkamp G.-J., Verpoorte R., Choi Y.H.	Natural deep eutectic solvents as a new extraction media for phenolic metabolites in carthamus tinctorius L.	475
Ruesgas-Ramon M., Figueroa-Espinoza M.C., Durand E.	Application of Deep Eutectic Solvents (DES) for Phenolic Compounds Extraction: Overview, Challenges, and Opportunities	402

Kumar A.K., Parikh B.S., Pravakar M.	Natural deep eutectic solvent mediated pretreatment of rice straw: bioanalytical characterization of lignin extract and enzymatic hydrolysis of pretreated biomass residue	384
Cunha S.C., Fernandes J.O.	Extraction techniques with deep eutectic solvents	374
Satlewal A., Agrawal R., Bhagia S., Sangoro J., Ragauskas A.J.	Natural deep eutectic solvents for lignocellulosic biomass pretreatment: Recent developments, challenges and novel opportunities	275
Van Osch D.J.G.P., Dietz C.H.J.T., Van Spronsen J., Kroon M.C., Gallucci F., Van Sint Annaland M., Tuinier R.	A Search for Natural Hydrophobic Deep Eutectic Solvents Based on Natural Components	254
Dai Y., Verpoorte R., Choi Y.H.	Natural deep eutectic solvents providing enhanced stability of natural colorants from safflower (Carthamus tinctorius)	251
Espino M., de los Angeles Fernandez M., Gomez F.J.V., Silva M.F.	Natural designer solvents for greening analytical chemistry	249
Dai Y., Rozema E., Verpoorte R., Choi Y.H.	Application of natural deep eutectic solvents to the extraction of anthocyanins from Catharanthus roseus with high extractability and stability replacing conventional organic solvents	248

Huang Y., Feng F., Jiang J., Qiao Y., Wu T., Voglmeir J., Chen Z.-G.	Green and efficient extraction of rutin from tartary buckwheat hull by using natural deep eutectic solvents	235
Vanda H., Dai Y., Wilson E.G., Verpoorte R., Choi Y.H.	Green solvents from ionic liquids and deep eutectic solvents to natural deep eutectic solvents	234
Craveiro R., Aroso I., Flammia V., Carvalho T., Viciosa M.T., Dionisio M., Barreiros S., Reis R.L., Duarte A.R.C., Paiva A.	Properties and thermal behavior of natural deep eutectic solvents	230
Cvjetko Bubalo M., Vidovic S., Radojcic Redovnikovic I., Jokic S.	New perspective in extraction of plant biologically active compounds by green solvents	222
Radosevic K., Curko N., Gaurina Srcek V., Cvjetko Bubalo M., Tomasevic M., Kovacevic Ganic K., Radojcic Redovnikovic I.	Natural deep eutectic solvents as beneficial extractants for enhancement of plant extracts bioactivity	213
Patzold M., Siebenhaller S., Kara S., Liese A., Syldatk C., Holtmann D.	Deep Eutectic Solvents as Efficient Solvents in Biocatalysis	212

OBS.: The most cited articles guide us towards what has already been consolidated as a reference on the subject and which cannot be missing from a review.

Table S7. Countries that Publish the Most

**S7. Countries that Publish the Most**			
**WoS**		**Scopus**	
**Country**	**Number of Articles**	**Country**	**Number of Articles**
China	226	China	249
Spain	90	Spain	94

United States	81	Italy	85
Italy	74	India	71
Canada	72	United States	65
India	65	Portugal	58
Portugal	56	Indonesia	55
Netherlands	46	Iran	46
Brazil	43	Netherlands	45
France	43	Malaysia	41
Iran	41	France	40
Indonesia	35	Poland	38
Poland	34	Brazil	37
Argentina	33	Argentina	34
Malaysia	32	Turkey	29
Croatia	29	Croatia	26
Turkey	29	Saudi Arabia	26
Saudi Arabia	27	Russian Federation	25
Germany	26	Serbia	22
Serbia	25	Germany	21
Russian Federation	24	Greece	21

OBS.: The countries that publish the most can point out future places for partnerships, or places where the theme is more present, important and addressed.

Table S8. Universities that Publish the Most

**S8. Universities that Publish the Most**			
**WoS**		**Scopus**	
**Affiliation Name**	**Number of Articles**	**Affiliation Name**	**Number of Articles**
UNIVERSITY OF TORONTO	68	LAQV-REQUIMTE	32
HOSPITAL FOR SICK CHILDREN SICKKIDS	67	Universiteit Leiden	31

LEIDEN UNIVERSITY	30	Universitas Indonesia	31
LEIDEN UNIVERSITY EXCL LUMC	29	Universidad Nacional de Cuyo	29
UNIVERSITY NACIONAL CUYO MENDOZA	26	Institute of Biology Leiden	28
UNIVERSITY OF INDONESIA	26	Instituto de Biología Agrícola de Mendoza	27
UNIVERSITY OF ZAGREB	26	Ministry of Education China	26
UNIVERSIDADE NOVA DE LISBOA	25	Faculdade de Ciências e Tecnologia da Universidade Nova de Lisboa	26
CONSEJO NACIONAL DE INVESTIGACIONES CIENTIFICAS Y TECNICAS CONICET	22	University of Zagreb	23
SOUTH CHINA UNIVERSITY OF TECHNOLOGY	21	South China University of Technology	22
UNIVERSIDAD DE BURGOS	17	Universidade do Minho	21
UNIVERSIDADE DO MINHO	16	Universidad de Burgos	19
UNIVERSITY OF TURIN	16	University of Zagreb, Faculty of Food Technology and Biotechnology	18
CONSEJO SUPERIOR DE INVESTIGACIONES CIENTIFICAS CSIC	15	Consejo Nacional de Investigaciones Científicas y Técnicas	17
UNIVERSITY OF NOVI SAD	15	Università degli Studi di Torino	17
UNIVERSITY OF PERUGIA	15	Zhejiang University of Technology	16
ZHEJIANG UNIVERSITY OF TECHNOLOGY	15	Universidade Nova de Lisboa	16
RUSSIAN ACADEMY OF SCIENCES	13	University of Novi Sad	16
UNIVERSITI MALAYA	13	Università degli Studi di Perugia	15
CENTRE NATIONAL DE LA RECHERCHE SCIENTIFIQUE CNRS	12	Consejo Superior de Investigaciones Científicas	14
CONSIGLIO NAZIONALE DELLE RICERCHE CNR	12	Sapienza Università di Roma	14
INRAE	12	Western Michigan University	13

OBS.: As well as authors and countries that publish the most, knowing the universities that are publishing the most on the topic addressed is important to redirect new researchers who intend to study the area, as well as those who seek partnerships.

Table S9. Agencies that Most Fund

S9. Agencies that Most Fund			
**WoS**		**Scopus**	
**Agencies that most fund**	**Number of Articles**	**Agencies that most fund**	**Number of Articles**
National Natural Science Foundation Of China Nsfc	110	National Natural Science Foundation Of China Nsfc	123
Spanish Government	64	Fundação para a Ciência e a Tecnologia	42
European Commission	44	European Regional Development Fund	30
Fundacao Para A Ciencia E A Tecnologia Fct	44	Horizon 2020 Framework Programme	29
Canadian Institutes Of Health Research Cihr	37	Consejo Nacional de Investigaciones Científicas y Técnicas	27
Consejo Nacional De Investigaciones Cientificas Y Tecnicas Conicet	27	European Commission	26
Cystic Fibrosis Canada	23	Ministerio de Ciencia, Innovación y Universidades	23
Conselho Nacional De Desenvolvimento Cientifico E Tecnologico Cnpq	22	National Key Research and Development Program of China	23
Coordenacao De Aperfeicoamento De Pessoal De Nivel Superior Capes	20	Universidad Nacional de Cuyo	23
National Key R D Program Of China	18	Coordenação de Aperfeiçoamento de Pessoal de Nível Superior	22
Natural Sciences And Engineering Research Council Of Canada Nserc	18	Conselho Nacional de Desenvolvimento Científico e Tecnológico	21
Fundacao De Amparo A Pesquisa Do Estado De Sao Paulo Fapesp	16	Ministério da Ciência, Tecnologia e Ensino Superior	19
Natural Science Foundation Of Zhejiang Province	15	Fundação de Amparo à Pesquisa do Estado de São Paulo	17
European Research Council Erc	14	Department of Science and Technology, Ministry of Science and Technology, India	16
Cgiar	13	European Research Council	16
Department Of Science Technology India	13	Hrvatska Zaklada za Znanost	16
National Institutes Of Health Nih Usa	13	Natural Science Foundation of Zhejiang Province	16
United States Department Of Health Human Services	13	Ministarstvo Prosvete, Nauke i Tehnološkog Razvoja	15
Croatian Science Foundation	12	Ministry of Higher Education, Malaysia	15

Facultad De Ciencias Agrarias Universidad Nacional De Cuyo Mendoza Argentina	12	Universitas Indonesia	15

OBS.: The major funding agencies become important for those seeking partnership and continuity in extensive research in the area.

Table S10. Areas that Publish the Most on the Topic

**S10. Areas that Publish the Most on the Topic**			
**WoS**		**Scopus**	
**Research Areas**	**Number of Articles**	**Research Areas**	**Number of Articles**
Chemistry	538	Chemistry	554
Biochemistry Molecular Biology	176	Chemical Engineering	287
Engineering	173	Agricultural and Biological Sciences	248
Food Science Technology	166	Biochemistry, Genetics and Molecular Biology	239
Science Technology Other Topics	122	Environmental Science	188
Physics	95	Materials Science	166
Pharmacology Pharmacy	81	Pharmacology, Toxicology and Pharmaceutics	145
Plant Sciences	61	Physics and Astronomy	134
Environmental Sciences Ecology	52	Engineering	100
Biotechnology Applied Microbiology	46	Energy	91
Nutrition Dietetics	43	Medicine	65
Agriculture	38	Immunology and Microbiology	41
Materials Science	32	Multidisciplinary	18
Polymer Science	32	Social Sciences	18
Immunology	30	Computer Science	15
Energy Fuels	22	Health Professions	15
Integrative Complementary Medicine	18	Business, Management and Accounting	12
Cell Biology	13	Economics, Econometrics and Finance	8
Thermodynamics	13	Earth and Planetary Sciences	6
Spectroscopy	12	Mathematics	6

OBS.: This analysis helps direct research on the topic of interest, aimed at those who already have extensive research in the area and even those who may become potential areas of coverage for the topic.

Table S11. Most cited articles when complementing the search with the terms "Low Transition Temperature Mixtures", "LTTMs" and "Deep Eutectic Solvents"

Authors	Article Name	Journal Information
Abbott, A. P., Harris, R. C., Ryder, K. S., D'Agostino, C., Gladden, L. F., & Mantle, M. D.	Glycerol eutectics as sustainable solvent systems	Green Chemistry. 2011;13(1), 82-90
Alvarez-Vasco, C., Ma, R., Quintero, M., Guo, M., Geleynse, S., Ramasamy, K. K., ... & Zhang, X.	Unique low-molecular-weight lignin with high purity extracted from wood by deep eutectic solvents (DES): a source of lignin for valorization	Green chemistry. 2016;18(19), 5133-5141
Carriazo, D., Serrano, M. C., Gutiérrez, M. C., Ferrer, M. L., & del Monte, F.	Deep-eutectic solvents playing multiple roles in the synthesis of polymers and related materials	Chemical Society Reviews. 2012;41(14), 4996-5014
Durand, E., Lecomte, J., & Villeneuve, P.	From green chemistry to nature: The versatile role of low transition temperature mixtures	Biochimie. 2016;120, 119-123
El Achkar T., Fourmentin S., Greige-Gerges H.	Deep eutectic solvents: An overview on their interactions with water and biochemical compounds	Journal of Molecular Liquids. 2019;288:111028
Florindo, C., Oliveira, F. S., Rebelo, L. P. N., Fernandes, A. M., & Marrucho, I. M.	Insights into the synthesis and properties of deep eutectic solvents based on cholinium chloride and carboxylic acids	ACS Sustainable Chemistry & Engineering. 2014;2(10), 2416-2425
Francisco M., González A. S., de Dios S. L. G., Weggemans W., Kroon M. C.	Comparison of a low transition temperature mixture (LTTM) formed by lactic acid and choline chloride with choline lactate ionic liquid and the choline chloride salt: physical properties and vapour–liquid equilibria of mixtures containing water and ethanol	RSC advances. 2013;3(45):23553–61
Francisco M., van den Bruinhorst A., Zubeir L. F., Peters C. J., Kroon M. C.	A new low transition temperature mixture (LTTM) formed by choline chloride+ lactic acid: Characterization as solvent for CO2 capture	Fluid Phase Equilibria. 2013;340:77–84
Francisco, M., Van Den Bruinhorst, A., & Kroon, M. C.	New natural and renewable low transition temperature mixtures (LTTMs): screening as solvents for lignocellulosic biomass processing	Green chemistry. 2012;14(8), 2153-2157
Francisco, M., van den Bruinhorst, A., & Kroon, M. C.	Low-transition-temperature mixtures (LTTMs): A new generation of designer solvents	Angewandte Chemie international edition. 2013;52(11), 3074-3085

García G., Aparicio S., Ullah R., Atilhan M.	Deep eutectic solvents: physicochemical properties and gas separation applications	Energy & Fuels. 2015;29(4):2616–44
Gill I., Vulfson E.	Enzymic catalysis in heterogeneous eutectic mixtures of substrates	Trends in biotechnology. 1994;12(4):118–22
Gonzalez, A. S., Francisco, M., Jimeno, G., de Dios, S. L. G., & Kroon, M. C.	Liquid–liquid equilibrium data for the systems {LTTM+ benzene+ hexane} and {LTTM+ ethyl acetate+ hexane} at different temperatures and atmospheric pressure	Fluid Phase Equilibria. 2013;360, 54-62
Hansen B. B., Spittle S., Chen B., Poe D., Zhang Y., Klein J. M., et al.	Deep eutectic solvents: A review of fundamentals and applications	Chemical reviews. 2020;121(3):1232–85
Hou, X. D., Li, A. L., Lin, K. P., Wang, Y. Y., Kuang, Z. Y., & Cao, S. L.	Insight into the structure-function relationships of deep eutectic solvents during rice straw pretreatment	Bioresource technology. 2018;249, 261-267
Hussin, S. A. M., Varanusupakul, P., Shahabuddin, S., Hui, B. Y., & Mohamad, S.	Synthesis and characterization of green menthol-based low transition temperature mixture with tunable thermophysical properties as hydrophobic low viscosity solvent	Journal of Molecular Liquids. 2020;308, 113015
Jancheva, M., Grigorakis, S., Loupassaki, S., & Makris, D. P.	Optimised extraction of antioxidant polyphenols from Satureja thymbra using newly designed glycerol-based natural low-transition temperature mixtures (LTTMs)	Journal of applied research on medicinal and aromatic plants. 2017;6, 31-40
Li, C., Li, D., Zou, S., Li, Z., Yin, J., Wang, A., ... & Zhao, Q.	Extraction desulfurization process of fuels with ammonium-based deep eutectic solvents	Green Chemistry. 2013;15(10), 2793-2799
Liu, P., Hao, J. W., Mo, L. P., & Zhang, Z. H.	Recent advances in the application of deep eutectic solvents as sustainable media as well as catalysts in organic reactions	RSC advances. 2015;5(60), 48675-48704
Manousaki, A., Jancheva, M., Grigorakis, S., & Makris, D. P.	Extraction of antioxidant phenolics from agri-food waste biomass using a newly designed glycerol-based natural low-transition temperature mixture: A comparison with conventional eco-friendly solvents	Recycling. 2016;1(1), 194-204.
Martins, M. A., Pinho, S. P., & Coutinho, J. A.	Insights into the nature of eutectic and deep eutectic mixtures	Journal of Solution Chemistry. 2019;48, 962-982
Mota-Morales, J. D., Sánchez-Leija, R. J., Carranza, A., Pojman, J. A., del Monte, F., & Luna-Bárcenas, G.	Free-radical polymerizations of and in deep eutectic solvents: Green synthesis of functional materials	Progress in Polymer Science. 2018;78, 139-153

Passos H., Tavares D. J., Ferreira A. M., Freire M. G., Coutinho J. A.	Are aqueous biphasic systems composed of deep eutectic solvents ternary or quaternary systems?	ACS Sustainable Chemistry & Engineering. 2016;4(5):2881–6
Rodriguez N. R., Molina B. S., Kroon M. C.	Aliphatic+ ethanol separation via liquid–liquid extraction using low transition temperature mixtures as extracting agents	Fluid Phase Equilibria. 2015;394:71–82
Rodriguez, N. R., & Kroon, M. C.	Isopropanol dehydration via extractive distillation using low transition temperature mixtures as entrainers	The Journal of Chemical Thermodynamics. 2015;85, 216-221
Rodríguez, N. R., González, A. S., Tijssen, P. M., & Kroon, M. C.	Low transition temperature mixtures (LTTMs) as novel entrainers in extractive distillation	Fluid Phase Equilibria. 2015;385, 72-78
Ruß, C., & König, B.	Low melting mixtures in organic synthesis–an alternative to ionic liquids?	Green Chemistry. 2012;14(11), 2969-2982
Sheldon, R. A.	The E factor 25 years on: the rise of green chemistry and sustainability	Green Chemistry. 2017;19(1), 18-43
Sutton, A. T., Fraige, K., Leme, G. M., da Silva Bolzani, V., Hilder, E. F., Cavalheiro, A. J., ... & Funari, C. S.	Natural deep eutectic solvents as the major mobile phase components in high-performance liquid chromatography—searching for alternatives to organic solvents	Analytical and bioanalytical chemistry. 2018;410, 3705-3713
Tang, S., Baker, G. A., & Zhao, H.	Ether-and alcohol-functionalized task-specific ionic liquids: attractive properties and applications	Chemical Society Reviews. 2012;41(10), 4030-4066
Tang, X., Zuo, M., Li, Z., Liu, H., Xiong, C., Zeng, X., ... & Lin, L.	Green processing of lignocellulosic biomass and its derivatives in deep eutectic solvents	ChemSusChem. 2017;10(13), 2696-2706
Tereshatov, E. E., Boltoeva, M. Y., & Folden, C. M.	First evidence of metal transfer into hydrophobic deep eutectic and low-transition-temperature mixtures: indium extraction from hydrochloric and oxalic acids	Green Chemistry. 2016;18(17), 4616-4622
Tian D., Chandra R. P., Lee J. S., Lu C., Saddler J. N.	A comparison of various lignin-extraction methods to enhance the accessibility and ease of enzymatic hydrolysis of the cellulosic component of steam-pretreated poplar	Biotechnology for biofuels. 2017;10:1–10
van Osch, D. J., Zubeir, L. F., van den Bruinhorst, A., Rocha, M. A., & Kroon, M. C.	Hydrophobic deep eutectic solvents as water-immiscible extractants	Green Chemistry. 2015;17(9), 4518-4521
Wagle D. V., Zhao H., Baker G. A.	Deep eutectic solvents: sustainable media for nanoscale and functional materials	Accounts of chemical research. 2014;47(8):2299–308
Yiin C. L., Quitain A. T., Yusup S., Sasaki M., Uemura Y., Kida T.	Characterization of natural low transition temperature mixtures (LTTMs): Green solvents for biomass delignification	Bioresource technology. 2016;199:258–64

Yiin, C. L., Quitain, A. T., Yusup, S., Uemura, Y., Sasaki, M., & Kida, T.	Sustainable green pretreatment approach to biomass-to-energy conversion using natural hydro-low-transition-temperature mixtures	Bioresource technology. 2018;261, 361-369
Yiin, C. L., Yusup, S., Quitain, A. T., Uemura, Y., Sasaki, M., & Kida, T.	Thermogravimetric analysis and kinetic modeling of low-transition-temperature mixtures pretreated oil palm empty fruit bunch for possible maximum yield of pyrolysis oil	Bioresource technology. 2018;255, 189-197
Yin J., Wang J., Li Z., Li D., Yang G., Cui Y., et al.	Deep desulfurization of fuels based on an oxidation/extraction process with acidic deep eutectic solvents	Green Chemistry. 2015;17(9):4552–9
Yu, D., Xue, Z., & Mu, T.	Eutectics: formation, properties, and applications	Chemical Society Reviews. 2021;50(15), 8596-8638
Zubeir, L. F., Lacroix, M. H., & Kroon, M. C.	Low transition temperature mixtures as innovative and sustainable CO2 capture solvents	The Journal of Physical Chemistry B. 2014;118(49), 14429-14441
